# Influence of Temperature, Humidity, and Photophase on the Developmental Stages of *Spodoptera litura* (Lepidoptera: Noctuidae) and Prediction of Its Population Dynamics

**DOI:** 10.3390/insects16040355

**Published:** 2025-03-27

**Authors:** Chun Fu, Zhiqian Liu, Danping Xu, Tingjiang Gan, Xinqi Deng, Honghua Zhang, Zhihang Zhuo

**Affiliations:** 1 Key Laboratory of Sichuan Province for Bamboo Pests Control and Resource Development, Leshan Normal University, Leshan 614000, China; fuchun421@aliyun.com; 2 College of Life Science, China West Normal University, Nanchong 637002, China; qnhtvxhp319123@foxmail.com (Z.L.); xudanping@cwnu.edu.cn (D.X.); deng.xinqi@foxmail.com (X.D.); honghua_zhang@foxmail.com (H.Z.); 3 Engineering Research Centre of Chuanxibei Rural Human Settlement (RHS) Construction, Mianyang Teachers’ College, Mianyang 621016, China; gantinjiangky@mtc.edu.cn

**Keywords:** *Spodoptera litura*, climate change, agricultural pests, invasive insects, meta-analysis

## Abstract

*Spodoptera litura* (Fabricius, 1775) is a major agricultural pest that primarily targets vegetables, cash crops, peanuts, and sugarcane. It causes damage to leaves, flower buds, and fruits, leading to significant reductions in crop yields. Global climate change may profoundly affect the population dynamics and biological traits of this pest. This research employs a meta-analysis to systematically investigate the impact of temperature variation on the developmental parameters of *S. litura*. A detailed review of 17 relevant studies reveals that within an optimal temperature range (30 °C to 35 °C), higher temperatures expedite the developmental processes of *S. litura*, shorten its life cycle, and enhance the reproductive potential of female adults. In contrast, temperatures exceeding 35 °C slow down its development, increase mortality rates, and markedly reduce the egg-laying capacity of females, highlighting the adverse effects of heat stress on growth and reproduction. Furthermore, different life stages of *S. litura* exhibit varying degrees of temperature sensitivity, with the larval stage being particularly vulnerable to high temperatures, while extreme heat significantly suppresses adult survival. These meta-analysis findings shed light on the biological responses of *S. litura* to climate change and provide a scientific basis for developing future pest management strategies. As global temperatures rise, moderate warming may facilitate the spread of *S. litura* populations, exacerbating their threat to crop production, whereas extreme heat conditions could constrain their growth. Consequently, pest control strategies must be more region-specific and aligned with local climatic trends.

## Simple Summary

*Spodoptera litura* (Fabricius, 1775) is a significant economic pest that has successfully invaded Africa and Asia in recent years. This study systematically evaluated the life history traits of *S. litura* under different temperature, photoperiod, and humidity conditions. The results showed that at 30–35 °C, the physiological activity of *S. litura* peaked, with a significant reduction in the duration of developmental stages, increased female oviposition, shortened pupal and adult lifespans, and an accelerated generational cycle. These findings provide critical insights into predicting population dynamics and offer valuable guidance for developing effective management strategies.

## 1. Introduction

Climate change has become one of the most challenging environmental issues facing modern agricultural ecosystems, profoundly influencing the occurrence and development of crop pests and diseases [1,2]. Changes in climate factors such as temperature, precipitation, and atmospheric CO_2_ concentration have not only altered the dynamics of agricultural ecosystems but also had far-reaching impacts on pest growth and development, reproductive capacity, migration behavior, and biodiversity [3,4,5]. In this context, *S. litura*, as a major pest in global agricultural production, may experience significant changes in its biological characteristics due to the effects of climate change, potentially posing a threat to agricultural production [6,7]. Investigating the impact of climate change on the biological characteristics of *S. litura* is crucial for developing effective pest control strategies.

*Spodoptera litura* (Fabricius, 1775) is widely distributed across agricultural regions globally, particularly in tropical and subtropical areas, where its larvae primarily feed on crops, especially maize, soybeans, and cotton [8]. As a polyphagous pest active both day and night, *S. litura* can adapt to various climatic conditions, and its growth, development, reproductive capacity, and migration behaviors are highly sensitive to climate change [9]. In recent years, with the intensification of global climate change, the distribution and frequency of *S. litura* occurrences have expanded, posing an increasing challenge to agricultural production [10]. Climate change impacts *S. litura*’s development rate, reproductive potential, survival rate, and population dynamics, directly or indirectly altering its biological characteristics [11,12]. Therefore, systematically assessing the combined effects of climate factors, particularly changes in temperature and humidity, on *S. litura* will provide scientific evidence for early pest warning systems and effective pest control strategies.

Existing studies have shown that temperature is a key factor influencing the biological characteristics of *S. litura* [13,14]. Higher temperatures typically accelerate its life cycle, shorten the duration of each developmental stage, increase generation turnover, and lead to population outbreaks [15]. However, excessively high temperatures may exceed the species’ adaptive capacity, resulting in increased mortality or incomplete development [16]. Additionally, humidity and photoperiod may significantly impact the survival and distribution of *S. litura* [17]. Droughts or extreme precipitation events not only affect habitat suitability but may also alter the availability of food resources [18]. Therefore, comprehensively assessing the impact of these climate factors on *S. litura* will help provide more targeted strategies for pest management.

Although previous studies have explored the effects of climate change on the biological characteristics of *S. litura*, there are some discrepancies and uncertainties due to differences in research methods, climate scenarios, and regional contexts. Meta-analysis, as an effective statistical approach, can integrate the results of different studies, quantify the overall impact of climate change on the biological characteristics of *S. litura*, and reveal the magnitude and direction of the effects of various climate factors [19]. This study uses meta-analysis to address the following questions: How does climate change affect the development rate, reproductive potential, and survival rate of *S. litura*? The findings will not only contribute to a deeper understanding of the ecological responses of *S. litura* under climate change but also provide data support and theoretical foundations for future pest prediction models and control strategies.

## 2. Materials and Methods

### 2.1. Literature Search

In this study, we employed a systematic literature review approach, gathering relevant research from various databases [20]. The literature search was conducted between August and October 2024, primarily focusing on Web of Science, PubMed, Scopus, and CNKI. Additionally, the reference lists of related review articles were manually examined by us to identify studies not indexed in these databases. The search terms included “*Spodoptera litura*”, “climate change” (including “global warming” or “climate change”), “temperature”, “humidity” (or “precipitation”), and “biological traits” (such as “biological traits”, “life history traits”, “development”, or “reproduction”). Boolean operators (AND, OR) were used to ensure comprehensive coverage of the relevant literature [21].

The selection process was conducted in two steps. In the first step, titles and abstracts were screened to exclude studies unrelated to climate change or *S. litura*. In the second step, a full-text review was performed to exclude studies that did not meet the inclusion criteria. The inclusion criteria required the study to explicitly investigate the effects of climate factors, such as temperature and humidity, on biological traits of *S. litura* (e.g., developmental rate, reproductive capacity, survival rate). Studies were also required to include a control group and provide clear documentation of experimental conditions (e.g., temperature range, humidity levels). Additionally, the study had to provide data suitable for meta-analysis (e.g., means, standard deviations, sample sizes) or data that could be extracted from figures. Exclusion criteria included studies lacking a control group, those based solely on theoretical models, studies unrelated to *S. litura* biological traits or climate factors, and studies that did not provide sufficient statistical data for meta-analysis.

### 2.2. Data Extraction

Data were gathered from studies that met the inclusion criteria. The key variables included temperature (experimental treatment temperatures in °C), relative humidity, and biological traits (such as development rate, generation time, and reproductive metrics like egg-laying ability). For studies lacking direct statistical details, such as standard deviations or sample sizes, the required data were retrieved from figures using graphical extraction tools like WebPlotDigitizer (v5). In cases of multivariate experiments or studies with multiple treatment groups, each treatment was treated as an independent effect size for analysis.

### 2.3. Statistical Analysis

The analysis was conducted using the “rma.mv” function from the “metafor” package in R version 4.3 [22]. A random-effects model was applied to calculate the relative risk (RR+) and estimate variance (I^2^) between cases using restricted maximum likelihood (REML) [23,24,25]. Explanatory variables were added to the model based on the I^2^ value. The overall mean effect size of temperature across all treatment groups was determined using a random-effects model [26]. Statistical tests, including mean effect size, 95% confidence intervals (CI), Qt, and I^2^, were performed. “Qt” is used to represent the total heterogeneity statistic (the overall value of Cochran’s Q) after combining all included studies, which helps to assess whether there are significant differences between the study results.

Meta-analyses were conducted to assess the impact of temperature on various biological traits of *S. litura* across different developmental stages. Both random-effects and fixed-effects models were used, with the random-effects model preferred due to significant heterogeneity in experimental locations and methods [27].

In this analysis, temperature and relative humidity were treated as independent variables, while the biological traits of *S. litura* were the dependent variables. Effect sizes were calculated using the Log Response Ratio (LRR) [28], with statistical significance assessed through 95% confidence intervals. Subgroup analyses were performed to examine the influence of temperature and relative humidity on *S. litura*’s biological traits.

Heterogeneity was assessed using Q statistics and the I^2^ index [29]. The I^2^ value indicates the extent of variability, with higher values signifying greater heterogeneity. The heterogeneity statistic was calculated by testing the weighted sum of squares based on a k-1 distribution. If the 95% confidence interval of the effect size includes 0, there is no significant difference between the experimental and control groups (*p* > 0.05). If the confidence interval is entirely above 0, the experimental group shows a significantly larger effect size (*p* < 0.05). Conversely, if the confidence interval is entirely below 0, the experimental group shows a smaller effect size (*p* < 0.05).

Explanatory variables were included based on the significance of the cumulative effect size relative to zero and the *p*-value of Qt. Key variables considered included the effects of humidity and photoperiod on the cumulative effect size. Temperature was treated as a continuous variable to assess its impact on the mean effect size. In the meta-analysis, heterogeneity was divided into between-group variance (explained by categorical factors) and within-group residual variance, with statistical significance assessed using a k-1 test. Publication bias was evaluated using funnel plots and radar charts [30,31]. If bias was detected, the “trim and fill” method was used to correct it [32].

## 3. Results

### 3.1. Literature Search and Screening Results

A comprehensive search identified 751 studies that could potentially be relevant. In the initial screening, 508 studies not related to the research topic were removed. After a thorough full-text review of the remaining 243 studies, 17 studies that fulfilled the inclusion criteria were selected. These studies investigated the impact of various climate factors, such as temperature and humidity, on the biological traits of *S. litura*. A total of 857 datasets were extracted from these 17 studies, covering the following parameters: 1st instar (*n* = 43), 2nd instar (*n* = 43), 3ird instar (*n* = 43), 4th instar (*n* = 43), 5th instar (*n* = 43), 6th instar (*n* = 43), adult longevity (female), *n* = 51), adult longevity (male), *n* = 47), adult stage (*n* = 26), egg (*n* = 105), egg-to-adult (*n* = 16), generation (*n* = 28), oviposition (*n* = 8), pre-oviposition (*n* = 35), pre-pupa (*n* = 15), pupa (*n* = 116), fertility (*n* = 31), and larval stage (*n* = 121). In addition, the overall impact of temperature changes on *S. litura* was analyzed (Table 1).

### 3.2. Overall Effect of Temperature on the Biological Traits of S. litura

**Figure 1:**
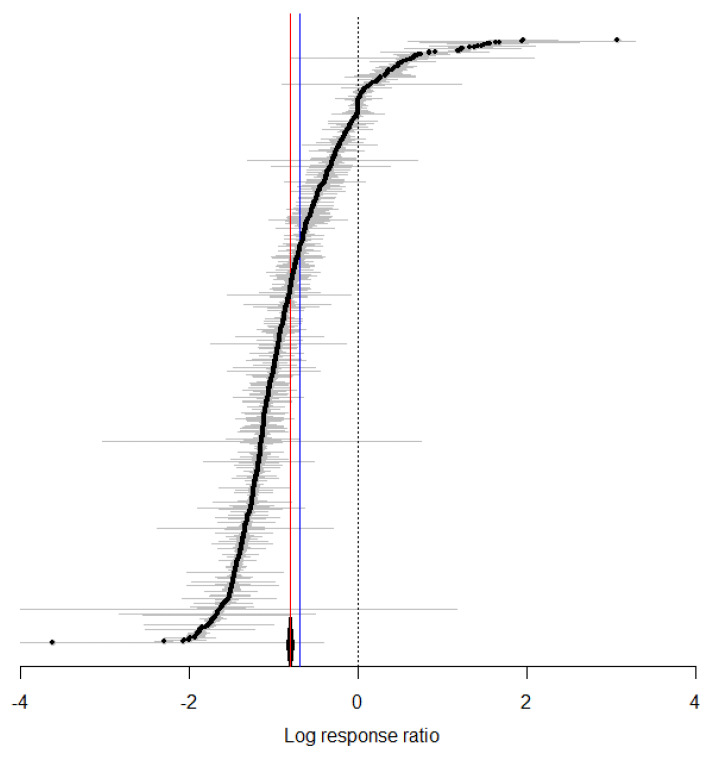
The effect of temperature changes on *S. litura* is presented. (Red line indicates the result derived from the random-effects model, showing a total effect size of −0.8077 with a 95%CI ranging from −0.8509 to −0.7645. In comparison, Black solid line represents the fixed-effects model result, with a total effect size of −0.6888 and a 95%CI of −0.6898 to −0.6878. The blue solid line shows the size of the cumulative effect).

**Figure 2:**
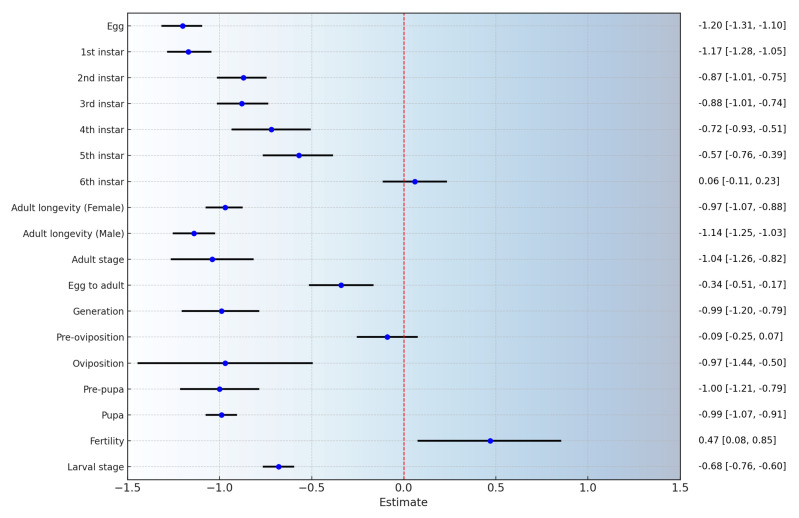
The effect of temperature changes on various physiological parameters of *S. litura*. (Blue dots represent the total effect size, and the black solid lines represent the 95% upper and lower confidence intervals).

**Figure 3:**
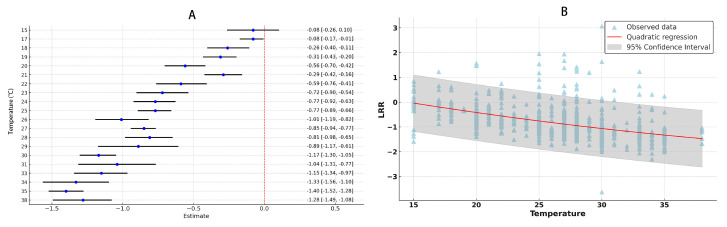
The impact of temperature changes on the developmental rate of *S. litura* is illustrated. Panel (**A**) shows how the physiological characteristics of *S. litura* vary with increasing temperature. The magnitude of the mean value reflects the impact of temperature: smaller mean values indicate a faster developmental rate, while larger mean values suggest a slower rate. (**B**) presents the temperature range curve that identifies the optimal growth conditions for *S. litura*.

After synthesizing the data from various studies and performing a meta-analysis, the findings suggest that increasing temperature increases the adaptability of *S. litura*. The overall mean effect size is −0.8077 (CI: −0.8509; −0.7645; Figure 1, Table 2, Figure S1). As temperature rises, the duration of the oviposition period decreases, the oviposition rate of female adults increases, egg hatching time shortens, the development period of 1st to 5th instar larvae is reduced, and adult longevity increases. However, there is no significant effect on the development period of the 6th instar larvae or the pre-oviposition period (Figure 2). By considering temperature as a continuous factor, the overall impact of temperature variations on *S. litura* across different temperature ranges was observed. With rising temperature, all physiological parameters of *S. litura* significantly increased (Figure 3A). As the temperature rises, the physiological activity of *S. litura* peaks at 35 °C. However, when temperatures exceeded 35 °C, there was a noticeable decline in all physiological indicators (Figure 3B).

### 3.3. The Impact of Temperature on Development Period

**Figure 4:**
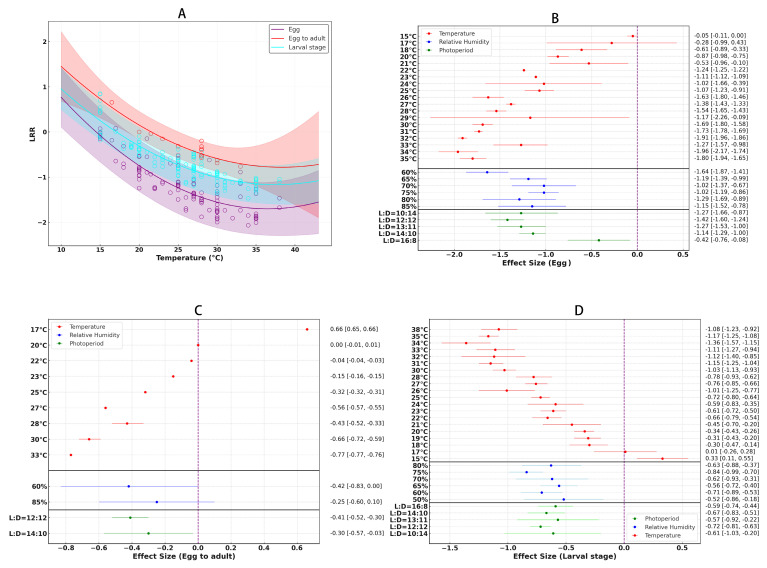
The effects of temperature on the developmental duration of *S. litura* eggs, egg-to-adult development, and larval stage are shown. Panel (**A**) illustrates the trend in developmental time as temperature increases. Panels (**B**−**D**) highlight the optimal environmental conditions for the development of eggs, egg-to-adult stages, and larvae, respectively.

Temperature has a significant impact on the hatching rate and developmental speed of *S. litura* eggs (Figure 4A), with an overall average effect size of −1.2022 (CI: −1.3081; −1.0963; Figure S2, Table 2). The temperature–response curve analysis revealed that 34 °C is the optimal temperature for egg development (Figure 4B). At this temperature, the hatching rate is highest, and the developmental speed is fastest, indicating that 34 °C provides the ideal conditions for egg growth. Both higher and lower temperatures result in a marked decline in hatching rate and developmental speed. Additionally, the optimal humidity for the egg stage was found to be 60%, with this relatively low humidity being sufficient to support normal egg development. Regarding photoperiod, the optimal light cycle for the egg stage was determined to be 12 h light:12 h dark, suggesting that *Spodoptera litura* eggs develop most effectively under a 12:12 light–dark cycle.

For the egg to adult developmental stage, temperature has a significant impact on the hatching rate and developmental speed of *S. litura* egg to adult (Figure 4A), with an overall average effect size of −0.3387 (CI: −0.5102; −0.1672; Figure S3, Table 2). the optimal temperature slightly decreases to 33 °C (Figure 4C). At this temperature, development from egg to adult proceeds most smoothly, indicating that the temperature requirement for this stage is slightly lower than that of the egg stage. When temperatures deviate from this value, development is significantly inhibited, leading to lower hatching and survival rates. Humidity for this stage was also found to be 60%, similar to the egg stage, which effectively supports normal developmental processes. The optimal photoperiod for the egg to adult stage was also 12:12, consistent with the egg stage, highlighting the importance of the 12 h light and 12 h dark cycle for proper development during this phase.

Temperature continues to play a crucial role during the larval stage of *S. litura* (Figure 4A), with an overall average effect size of −0.6795 (CI: −0.7630; −0.5960; Figure S4, Table 2). The optimal temperature for larval development is 34 °C, providing the best conditions for growth and survival (Figure 4D). Similar to the egg stage, any deviation from 34 °C leads to a significant reduction in developmental speed and survival rates. In terms of humidity, the optimal level for the larval stage was notably higher than in the other stages, reaching 75%, which facilitates better growth and survival. This higher humidity level is critical for supporting healthy larval development. Like the egg and egg to adult stages, the larval stage also thrives under a 12:12 light cycle, indicating a strong dependence on a consistent photoperiod for proper growth and development.

### 3.4. Temperature Effects on Developmental Duration at Different Larval Stages

**Figure 5:**
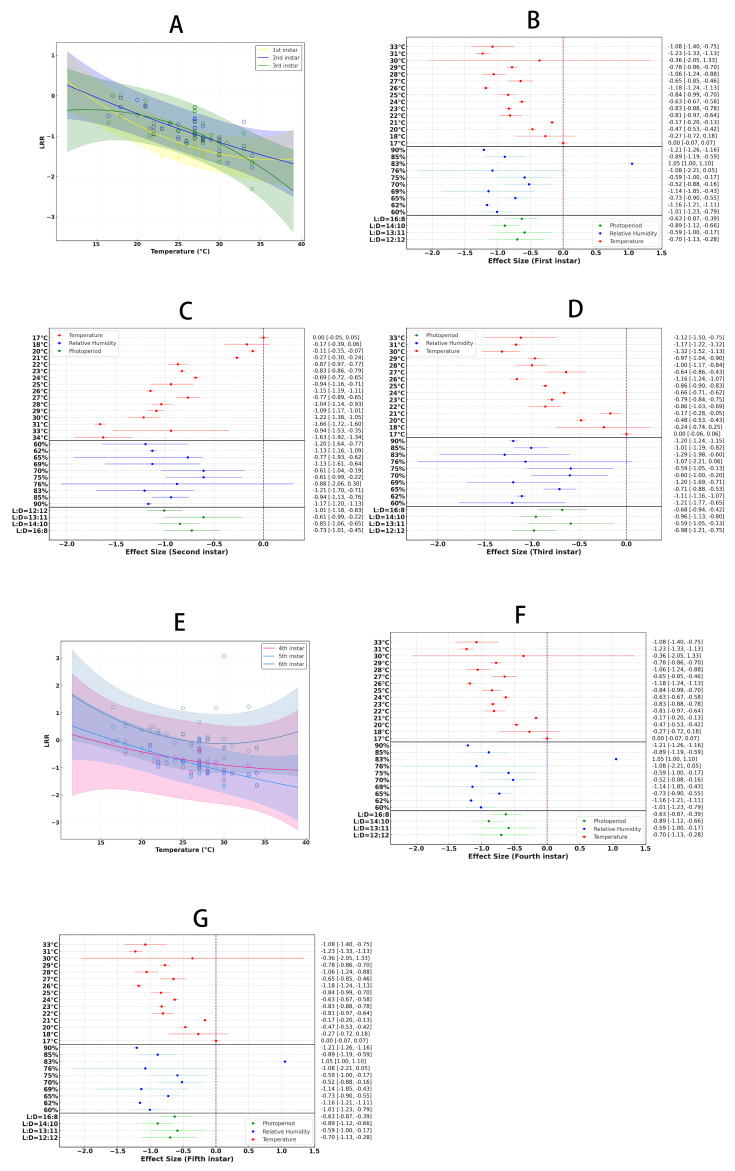
The impact of temperature on various developmental stages of *S. litura* is demonstrated. Panel (**A**) shows how the developmental time of first-to-third instar larvae changes with temperature variations, while Panels (**B**∓**D**) depict their response to external environmental conditions. Similarly, Panel (**E**) illustrates the changes in developmental time of fourth-to-sixth instar larvae with temperature variations, and Panels (**F**,**G**) present the response of fourth-to-fifth instar larvae to external environmental factors.

In the first instar of *Spodoptera litura*, temperature significantly influenced development, as shown by the temperature response curve analysis (Figure 5A), with an overall average effect size of −1.1675 (CI: −1.2839; −1.0512; Figure S5, Table 2). The optimal temperature for development was 33 °C (Figure 5B). At this temperature, the first instar larvae exhibited the fastest development and best growth. Temperatures that were either too high or too low resulted in delayed development and decreased survival rates, making 33 °C the ideal temperature for this stage. Additionally, a humidity of 76% provided an optimal environment for development, and a 14:10 light:dark cycle offered appropriate light exposure and darkness, promoting healthy larval growth.

For the second instar, temperature also had a significant impact on development (Figure 5A), with an overall average effect size of −0.8725 (CI: −0.9938; −0.7512; Figure S6, Table 2). The optimal growth temperature was 31 °C (Figure 5C). Compared to the first instar, the second instar larvae were more adapted to slightly lower temperatures, and a humidity level of 76% supported normal development. The ideal light cycle was 12:12 (12 h light:12 h dark), which provided the appropriate diurnal variation necessary for optimal growth and development during this stage.

Temperature had a similarly significant effect on the development of the third instar (Figure 5A), with an overall average effect size of −0.8763 (CI: −1.0132; −0.7393; Figure S7, Table 2). The optimal temperature for the third instar was 30 °C (Figure 5D). Compared to the previous instars, the temperature was slightly lower, and the humidity increased to 83%, which contributed to faster growth and higher survival rates. The light cycle remained at 12:12, continuing to play a crucial role in promoting development. At this stage, larvae exhibited a significantly higher demand for humidity, and the temperature of 30 °C proved to be the most suitable for their growth, providing the optimal developmental conditions.

In the fourth instar, temperature remained a critical factor influencing development (Figure 5E), with an overall average effect size of −0.72 (CI: −0.9332; −0.5068; Figure S8, Table 2). Within the temperature range of 17–34 °C, the optimal growth temperature was 31 °C (Figure 5F). At this stage, the temperature slightly increased to 31 °C, and the humidity decreased slightly to 82%, yet remained at a relatively high level. Compared to the third instar, the fourth instar larvae demonstrated greater adaptability to the 12:12 light: dark cycle, which continued to be the optimal photoperiod for growth and development. The combination of temperature and humidity provided ideal developmental conditions, ensuring efficient growth.

In the case of the fifth instar, temperature increase resulted in a shortened developmental period, with an overall average effect size of −0.8143 (CI: −0.9701; −0.6586; Figure S9, Table 2). Within the 17–34 °C temperature range, the developmental time at the fifth instar stage significantly decreased with rising temperature (Figure 5E). Different humidity and photoperiod conditions affected the developmental rate of the fifth instar. The optimal conditions for the fifth instar were found at 30 °C, 76% relative humidity, and a photoperiod of 12:12 (L:D) (Figure 5G).

However, no significant effect of temperature increase was observed for the sixth instar stage, with an overall average effect size of 0.0597 (CI: −0.1070; 0.2263; Figure S10, Table 2). Within the temperature range of 17–34 °C, the developmental time for the sixth instar was not significantly affected by temperature. The cumulative effect sizes suggest that within this temperature range, temperature changes did not significantly influence the developmental time at the sixth instar stage.

### 3.5. The Effect of Temperature Changes on the Lifespan of Adult S. litura

**Figure 6:**
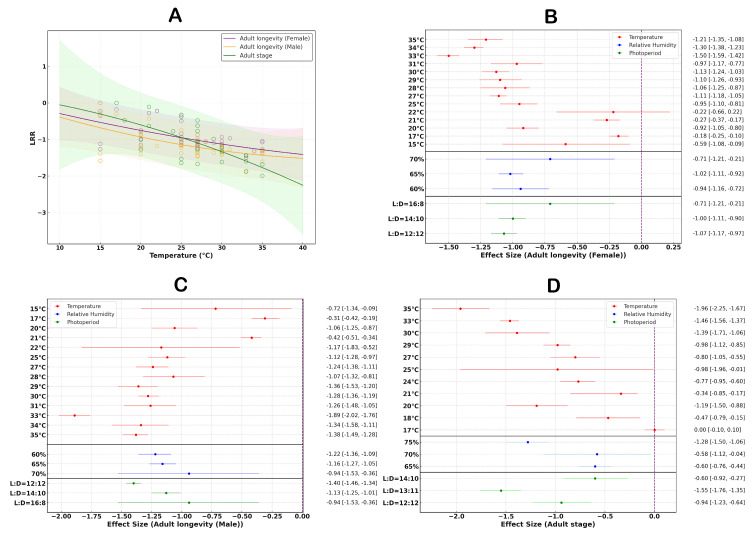
The effects of temperature on the lifespan of adult *S. litura.* (**A**) shows that the changes in the lifespan of female and male adults with increasing temperature. (**B**) depicts the response of female adult lifespan to variations in external environmental conditions. (**C**) depicts the response of male adult lifespan to variations in external environmental conditions. (**D**) depicts the response of the adult stage duration to variations in external environmental conditions.

Temperature significantly affects the lifespan of female *S. litura* (Figure 6A). The shortest lifespan for female adults occurs under conditions of 33 °C temperature, 65% humidity, and a 12:12 light cycle (Figure 6B). The temperature response curve indicates that at this temperature, the lifespan of female adults is significantly minimized. The overall average effect size is −0.9734 (CI: −1.0667; −0.8801; Figure S11, Table 2), suggesting that an increase in temperature significantly reduces the lifespan of female adults.

**Figure 7:**
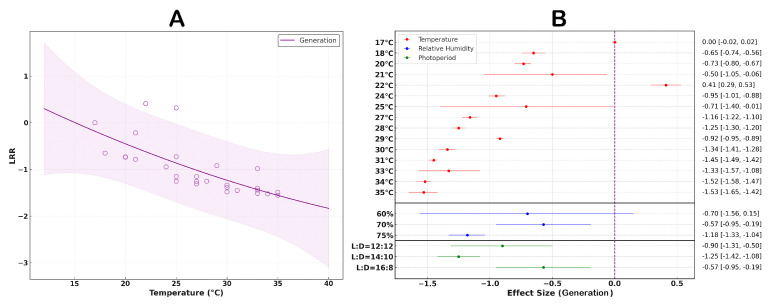
The influence of temperature variation on the life cycle of *S. litura*. (**A**) illustrates how the life cycle of *S. litura* changes with increasing temperature. (**B**) shows the response of the life cycle of *S. litura* to variations in external environmental conditions.

Similarly, temperature also has a significant impact on the lifespan of male *S. litura* adults (Figure 7A). The shortest lifespan for male adults occurs under conditions of 33 °C temperature, 60% humidity, and a 12:12 light cycle (Figure 6C). According to this temperature response curve, the lifespan of male adults reaches its minimum under these conditions. The overall average effect size is −1.1394 (CI: −1.2488; −1.03; Figure S12, Table 2), indicating that elevated temperatures have a significant negative effect on the lifespan of male adults.

Additionally, the studies indicate that an increase in temperature shortens the adult stage duration of *S. litura*, with an overall average effect size of −1.0392 (CI: −1.2611; −0.8173; Figure S13, Table 2). Within the temperature range of 17–35 °C, the adult stage duration significantly decreases as temperature rises (Figure 6A). The results show that the shortest adult stage duration occurs when the temperature reaches 35 °C, with relative humidity at 75%, and a light cycle of 13:11 (Figure 6D).

### 3.6. The Effect of Temperature on the Generation Cycle of S. litura

The studies indicate that an increase in temperature shortens the generation cycle of *S. litura*, with an overall average effect size of −0.9917 (CI: −1.1961; −0.7873; Figure S14). Within the temperature range of 17–35 °C, the generation cycle time significantly decreases as temperature rises (Figure 7A). The findings suggest that variations in the light cycle have a significant effect on the generation cycle time. The fastest generation cycle is observed when the temperature reaches 35 °C, with relative humidity at 75%, and a light cycle of 14:10 (Figure 7B).

### 3.7. Model Validation

**Figure 8:**
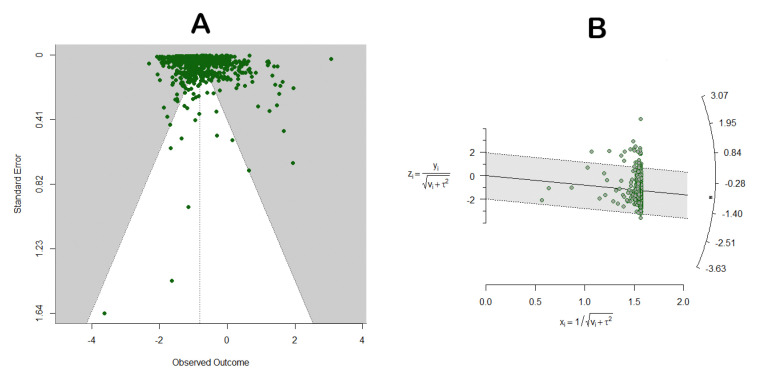
Funnel chart and radar chart. (**A**) shows a funnel chart, and (**B**) shows a radar chart.

Funnel plots and radar charts were utilized to assess whether publication bias influenced the results, and the failsafe number was calculated to verify the reliability of the findings. The results demonstrate that the funnel plot (Figure 8A, z = 11.7603, *p* = 0.0702), radar chart (Figure 8B), and failsafe number (*n* = 276385) all confirm the robustness of our results.

## 4. Discussion

As ectothermic organisms, insect populations are heavily influenced by temperature [33]. Global warming has been shown to significantly alter the spatial distribution of numerous insect species, leading to range expansion and regional shifts [34]. Temperature is thus a critical factor in the survival, development, and reproduction of *S. litura*. This study confirms that temperature profoundly impacts the growth and developmental cycle of this pest. Within an optimal temperature range, increasing temperatures significantly accelerate its growth and development. An analysis was conducted on the relationship between the developmental stages of *S. litura* and temperature, resulting in feedback curves that highlight temperature-dependent variations across all stages (Table 3). The findings indicate that *S. litura* thrives best at temperatures between 30–35 °C and relative humidity levels of 60–83%. Global warming is projected to drastically alter ecosystems, reshaping habitat structures and increasing insect prevalence and distribution, which may exacerbate agricultural damage. This investigation points out that under anticipated warming conditions, *S. litura* is likely to develop stronger adaptive capabilities, posing greater challenges to agricultural production.

Temperature significantly influences the biological traits of *S. litura*. This meta-analysis indicates that increased temperatures accelerate both the developmental rate and reproductive potential of *S. litura*. However, the positive effects of temperature diminish or may even be reversed under extreme heat conditions. This finding aligns with previous studies [35], indicating that within an optimal temperature range, *S. litura* can shorten its life cycle and increase population size. Notably, when temperatures exceed their upper thermal tolerance (typically above 35 °C), both development rates and survival decrease significantly. This is likely due to the adverse physiological effects of temperature stress [36], suggesting that extreme heat may inhibit the expansion of *S. litura* populations. The responses to temperature vary across different developmental stages. According to the results of the meta-analysis, the development rates of the first to fifth larval instars increase as temperature rises, although sensitivity to temperature differs among instars. The early larval stages, particularly the 1st and 2nd instars, are highly sensitive to temperature fluctuations, showing a marked increase in development rates at higher temperatures. However, these stages also experience increased mortality at elevated temperatures, reflecting the high survival pressure during the larval phase. In contrast, the impact of temperature on the 6th instar is relatively moderate, suggesting that the later instar develops greater thermal tolerance. This result aligns with previous studies [35], demonstrating that later stages exhibit stronger adaptability to environmental changes. Temperature also significantly affects the oviposition behavior of female adults. According to the meta-analysis, oviposition rates rise with increasing temperatures, particularly within the ideal range of 30–35 °C, where female adults achieve peak reproductive output. However, when temperatures exceed 35 °C, oviposition rates decline, indicating that high temperatures suppress reproductive capacity. This may result from negative effects on ovarian development or egg quality, consistent with the hypothesis of impaired reproductive functions under thermal stress [37]. Thus, while moderate warming may promote population growth, extreme heat could suppress reproduction under global warming scenarios. The influence of temperature on the developmental duration of *S. litura* exhibits clear stage-specific effects. Developmental periods shorten with increasing temperature, highlighting the role of temperature in accelerating growth. However, this effect shows a lag during the adult stage. The meta-analysis reveals cumulative effects of temperature across developmental stages: while higher temperatures accelerate early-stage development, they also negatively impact adult survival and behavior. At elevated temperatures, the lifespan of adults is markedly reduced, and the generation cycle speeds up, likely because of the suppressive influence of heat on metabolic energy processes and behavioral capacity. Consequently, while temperature positively regulates developmental periods, high temperatures may limit adult survival, potentially affecting long-distance migration and dispersal.

In this study, we examined the impact of temperature variation on the growth and development of *S. litura*. However, in addition to temperature, environmental factors such as humidity and light also play crucial roles in the developmental processes of this species. Firstly, humidity is one of the key environmental factors influencing insect growth and development. Changes in humidity not only directly affect the physiological functions of *S. litura*, but can also indirectly impact its reproductive capacity and survival rate by altering the water balance within the insect. Studies have shown that higher humidity tends to increase egg-hatching rates and larval survival in *S. litura*, suggesting that an optimal humidity environment is beneficial for its development. Secondly, light is another important factor influencing the growth and development of *S. litura*. As a nocturnal insect, *S. litura* is primarily active at night and seeks shelter during the day. Variations in light intensity and the circadian rhythm can affect its activity patterns, foraging behavior, and physiological processes. With the ongoing global warming, it is anticipated that the distribution of *S. litura* will increase significantly [38,39]. In a warming world, will the adaptability of *S. litura* improve? To address this question, we reviewed and analyzed 17 relevant studies using meta-analysis to evaluate the responses of *S. litura* to temperature changes. The results indicated that within the temperature range of 15–38 °C, the adaptability of *S. litura* improved progressively with increasing temperatures. This finding aligns with previous studies, further validating the reliability of the model. Given the potential for *S. litura* to cause severe agricultural damage and economic losses, monitoring its adaptability is essential [40]. This study predicts, through meta-analysis, that *S. litura* exhibits peak adaptability at temperatures between 30–35 °C These findings provide critical insights for early warning systems regarding the direction of insect invasions and suitable habitats, offering valuable information for future monitoring and forecasting efforts.

This meta-analysis included a systematic review and selection of 17 studies investigating the effects of temperature on the biological traits of *S. litura*. The studies primarily focused on regions where *S. litura* is most prevalent, including East Asia, Southeast Asia, South Asia, and West Africa. Most of the research utilized controlled laboratory experiments, with a few employing field trials. While temperature is widely recognized as a critical environmental factor influencing *S. litura*, the studies exhibited heterogeneity in experimental conditions, species, and reporting methods. To address this variability, we employed a random-effects model and conducted subgroup analyses to explore the specific impacts of different temperatures on the biological traits of *S. litura*. Global warming is expected to profoundly alter ecosystem functions and structures, leading to shifts in the distribution of biological habitats [41,42]. The increasing prevalence and spread of insects, along with the resultant damages, will significantly impact agricultural production. This study highlights that under future warming scenarios, the adaptability of *S. litura* is likely to strengthen. The findings provide policymakers with critical insights to develop effective pest management strategies, thereby mitigating the potential widespread economic losses caused by pests under a warming climate.

## 5. Conclusions

This study conducted a systematic evaluation of the effects of temperature fluctuations on the biological traits of *S. litura* within the context of global climate change, using a meta-analysis. The results indicated that within the optimal temperature range of 30 °C to 35 °C, increasing temperatures significantly accelerated the developmental rate of *S. litura*, shortened its life cycle, and enhanced the oviposition rate of female adults. However, under extremely high-temperature conditions (above 35 °C), the developmental rate slowed, mortality increased, and the reproductive capacity of female adults was significantly suppressed, suggesting an upper-temperature threshold beyond which *S. litura*’s biological processes are negatively impacted. In addition to temperature, other environmental factors such as humidity and light also influence the growth and development of *S. litura*. The interaction between these factors and temperature, alongside the implications of climate change, presents a complex dynamic for *S. litura* populations. As global temperatures rise, shifts in humidity and light conditions may exacerbate or mitigate the impact of temperature extremes, affecting the overall population dynamics and adaptive strategies of this species. Importantly, the sensitivity of *S. litura* to temperature changes varies across developmental stages, with the larval stage being the most sensitive to high temperatures. This suggests that managing temperature, humidity, and light conditions will be crucial for predicting future pest outbreaks and for developing effective control strategies.

## Figures and Tables

**Table 1:** Data volume of *S. litura* after screening. (TR represents the temperature range, CT represents the control group temperature, and *n* represents the sample size).

TR	CT	*n*	Variable
17–34 °C	17 °C	43	1st instar
17–34 °C	17 °C	43	2nd instar
17–34 °C	17 °C	43	3ird instar
17–34 °C	17 °C	43	4rth instar
17–34 °C	17 °C	43	5th instar
17–34 °C	17 °C	43	6th instar
15–35 °C	15 °C	51	Adult longevity (Female)
15–35 °C	15 °C	47	Adult longevity (male)
17–35 °C	17 °C	26	Adult stage
15–38 °C	15 °C	105	Egg
17–33 °C	17 °C	16	Egg to adult
17–35 °C	17 °C	28	Generation
17–33 °C	17 °C	8	Oviposition
17–34 °C	17 °C	35	Pre-oviposition
17–35 °C	17 °C	15	Pre-pupa
15–38 °C	15 °C	116	Pupa
17–35 °C	17 °C	31	Fertility
15–38 °C	15 °C	121	Larval stage
15–38 °C	15 °C	857	*Spodoptera litura*

**Table 2:** Results of subgroup analysis calculated using the random-effects model.

Variable	Estimate	SE	Z	*p*	CI.lb	CI.ub	Loglik	AIC	BIC
1st instar	−1.1675	0.0594	−19.6663	<0.0001	−1.2839	−1.0512	−19.9416	43.8832	47.3585
2nd instar	−0.8725	0.0619	−14.0993	<0.0001	−0.9938	−0.7512	−21.6789	47.3578	50.8331
3ird instar	−0.8763	0.0699	−12.5428	<0.0001	−1.0132	−0.7393	−26.8192	57.6384	61.1137
4th instar	−0.72	0.1088	−6.6187	<0.0001	−0.9332	−0.5068	−45.3916	94.7831	98.2584
5th instar	−0.8143	0.0795	−10.2486	<0.0001	−0.9701	−0.6586	−32.1914	68.3829	71.8582
6th instar	0.0597	0.0850	0.7018	0.4828	−0.1070	0.2263	−35.2275	74.4549	77.9303
Adult longevity (Female)	−0.9734	0.0476	−20.4557	<0.0001	−1.0667	−0.8801	−17.2454	38.4908	42.3148
Adult longevity (male)	−1.1394	0.0558	−20.441	<0.0001	−1.2488	−1.03	−20.7576	45.5151	49.1724
Adult stage	−1.0392	0.1132	−9.1778	<0.0001	−1.2611	−0.8173	−21.6633	47.3266	49.7644
Egg	−1.2022	0.054	−22.2472	<0.0001	−1.3081	−1.0963	−85.7709	175.5419	180.8306
Egg to adult	−0.3387	0.0875	−3.8707	0.0001	−0.5102	−0.1672	−5.4890	14.9779	16.3940
Generation	−0.9917	0.1043	−9.5097	<0.0001	−1.1961	−0.7873	−22.2479	48.4958	51.0874
Oviposition	−0.9705	0.2411	−4.0252	<0.0001	−1.4434	−0.4979	−7.2267	18.4534	18.3452
Pre−oviposition	−0.092	0.083	−1.1081	0.2678	−0.2546	0.0707	−24.5303	53.0607	56.1134
Pre−pupa	−1.0001	0.1071	−9.3409	<0.0001	−1.2099	−0.7902	−7.5383	19.0766	20.3547
Pupa	−0.9870	0.0432	−22.8742	<0.0001	−1.0716	−0.9025	−74.7748	153.5496	159.0394
Fertility	0.4685	0.1958	2.3926	0.0167	0.0847	0.8523	−46.5657	97.1315	99.9339
Larval stage	−0.6795	0.0426	−15.9436	<0.0001	−0.763	−0.596	−79.0335	162.0671	167.642
*Spodoptera litura*	−0.8077	0.022	−36.6432	<0.0001	−0.8509	−0.7645	−849.4961	1702.9922	1712.4967

**Table 3:** The optimal environmental conditions for each developmental stage of *S. litura*.

Developmental History	Ideal Survival Temperature	Ideal Relative Humidity for Survival	Optimal Light/Dark Duration for Survival
1st instar	33 °C	76%	L:D = 14:10
2nd instar	31 °C	76%	L:D = 12:12
3ird instar	30 °C	83%	L:D = 12:12
4th instar	31 °C	82%	L:D = 12:12
5th instar	30 °C	76%	L:D = 12:12
Adult longevity (Female)	33 °C	65%	L:D = 12:12
Adult longevity (male)	33 °C	60%	L:D = 12:12
Adult stage	35 °C	75%	L:D = 13:11
Egg	34 °C	60%	L:D = 12:12
Egg to adult	33 °C	60%	L:D = 12:12
Generation	35 °C	75%	L:D = 14:10
Pre-pupa	30 °C	65%	L:D = 13:11
Pupa	30 °C	75%	L:D = 12:12
Fertility	34 °C	75%	L:D = 14:10
Larval stage	34 °C	75%	L:D = 12:12
*Spodoptera litura*	30−35 °C	60–83%	−

## Data Availability

The data supporting the results are available in a public repository at: https://doi.org/10.6084/m9.figshare.28587818, accessed on 11 March 2025.

## Supplementary Materials

The following supporting information can be downloaded at: https://www.mdpi.com/article/10.3390/insects16040355/s1. Figure S1: Comparison of effect sizes between random-effects and fixed-effects models; Figure S2: Comparison of effect sizes between random-effects and fixed-effects models; Figure S3: Comparison of effect sizes between random-effects and fixed-effects models; Figure S4: Comparison of effect sizes between random-effects and fixed-effects models; Figure S5: Comparison of effect sizes between random-effects and fixed-effects models; Figure S6: Comparison of effect sizes between random-effects and fixed-effects models; Figure S7: Comparison of effect sizes between random-effects and fixed-effects models; Figure S8: Comparison of effect sizes between random-effects and fixed-effects models; Figure S9: Comparison of effect sizes between random-effects and fixed-effects models; Figure S10: Comparison of effect sizes between random-effects and fixed-effects models; Figure S11: Comparison of effect sizes between random-effects and fixed-effects models; Figure S12: Comparison of effect sizes between random-effects and fixed-effects models; Figure S13: Comparison of effect sizes between random-effects and fixed-effects models; Figure S14: Comparison of effect sizes between random-effects and fixed-effects models; Figure S15: Comparison of effect sizes between random-effects and fixed-effects models; Figure S16: Comparison of effect sizes between random-effects and fixed-effects models; Figure S17: Comparison of effect sizes between random-effects and fixed-effects models; Figure S18: Comparison of effect sizes between random-effects and fixed-effects models; Figure S19: Comparison of effect sizes between random-effects and fixed-effects models.

## References

[1] Haq M., Mia M.T., Rabbi M.F., Ali M.A. (2011). Incidence and severity of rice diseases and insect pests in relation to climate change. Climate Change and Food Security in South Asia.

[2] Hakala K., Hannukkala A., Huusela-Veistola E., Jalli M., Peltonen-Sainio P. (2011). Pests and diseases in a changing climate a major challenge for Finnish crop production. Agric. Food Sci..

[3] Skendžić S., Zovko M., Živković I.P., Lešić V., Lemić D. (2021). The impact of climate change on agricultural insect pests. Insects.

[4] Subedi B., Poudel A., Aryal S. (2023). The impact of climate change on insect pest biology and ecology: Implications for pest management strategies, crop production, and food security. J. Agric. Food Res..

[5] Sharma H.C. (2014). Climate change effects on insects: Implications for crop protection and food security. J. Crop Improv..

[6] Zakria M., Zaib M.S., Abbas K., Sarmad M., Zaka S.M., Noor-ul-Ane M. (2022). Influence of temperature on the development and reproduction of *Spodoptera litura* (Fabricius) on castor bean: Implications for its use as a trap crop. Arthropod-Plant Interact..

[7] Ahmad M., Arif M.I., Ahmad M. (2007). Occurrence of insecticide resistance in field populations of *Spodoptera litura* (Lepidoptera: Noctuidae) in Pakistan. Crop Prot..

[8] Ahmad M., Ghaffar A., Rafiq M. (2013). Host plants of leaf worm, *Spodoptera litura* (Fabricius) (Lepidoptera: Noctuidae) in Pakistan. Asian J. Agric. Biol..

[9] Lu Y., Yuan M., Gao X., Kang T., Zhan S., Wan H., Li J. (2013). Identification and validation of reference genes for gene expression analysis using quantitative PCR in *Spodoptera litura* (Lepidoptera: Noctuidae). PLoS ONE.

[10] Yoon S., Lee W. (2021). Methodological analysis of bioclimatic variable selection in species distribution modeling with application to agricultural pests (*Metcalfa pruinosa* and *Spodoptera litura*). Comput. Electron. Agric..

[11] Maharjan R., Hong S., Ahn J., Yoon Y., Jang Y., Kim J., Lee M., Park K., Yi H. (2023). Temperature and host plant impacts on the development of *Spodoptera litura* (Fabricius)(Lepidoptera: Noctuidae): Linear and nonlinear modeling. Insects.

[12] Miyashita K. (1971). Effects of constant and alternating temperatures on the development of *Spodoptera litura* F.: Lepidoptera: Noctuidae. Appl. Entomol. Zool..

[13] Rao M.S., Prasad T.V. (2020). Temperature based phenology model for predicting establishment and survival of *Spodoptera litura* (Fab.) on groundnut during climate change scenario in India. J. Agrometeorol..

[14] Zhong T., Gong L., Pan Y., Li J., Lu A., Liu L., Wu H., Zhao Z., Wang L. (2024). Performance of *Spodoptera litura* (Lepidoptera: Noctuidae) in responses to different amplitudes of alternating temperatures across permissive warm temperature regimes. J. Econ. Entomol..

[15] Zhu S., Lu Z., Chen L., Yu W., Zhang S. (2000). Effect of temperature and food on *Spodoptera litura* population. Ying Yong Sheng Tai Xue Bao J. Appl. Ecol..

[16] Pham T.A., Hwang S. (2020). High temperatures reduce nutrients and defense compounds against generalist *Spodoptera litura* F. in Rorippa dubia. Arthropod-Plant Interact..

[17] Karmakar P., Pal S. (2017). Influence of temperature on food consumption and utilization parameters of the common cutworm, *Spodoptera litura* (Fab)(Lepidoptera: Noctuidae). J. Entomol. Zool. Stud..

[18] Harvey J.A., Tougeron K., Gols R., Heinen R., Abarca M., Abram P.K., Basset Y., Berg M., Boggs C., Brodeur J. (2023). Scientists’ warning on climate change and insects. Ecol. Monogr..

[19] Hedges L.V., Gurevitch J., Curtis P.S. (1999). The meta-analysis of response ratios in experimental ecology. Ecology.

[20] Cooper C., Booth A., Varley-Campbell J., Britten N., Garside R. (2018). Defining the process to literature searching in systematic reviews: A literature review of guidance and supporting studies. Bmc Med. Res. Methodol..

[21] Dinet J., Favart M., Passerault J.M. (2004). Searching for information in an online public access catalogue (OPAC): The impacts of information search expertise on the use of Boolean operators. J. Comput. Assist. Learn..

[22] Viechtbauer W. (2010). Conducting meta-analyses in R with the metafor package. J. Stat. Softw..

[23] Borenstein M., Hedges L.V., Higgins J.P., Rothstein H.R. (2010). A basic introduction to fixed-effect and random-effects models for meta-analysis. Res. Synth. Methods.

[24] Corbeil R.R., Searle S.R. (1976). Restricted maximum likelihood (REML) estimation of variance components in the mixed model. Technometrics.

[25] Kosimov Z.O. (2024). Methods for estimating integrated variance: Jump robustness issues in high frequency time series. Econ. Math. Methods.

[26] Garamszegi L.Z., Markó G., Herczeg G. (2012). A meta-analysis of correlated behaviours with implications for behavioural syndromes: Mean effect size, publication bias, phylogenetic effects and the role of mediator variables. Evol. Ecol..

[27] Clark T.S., Linzer D.A. (2015). Should I use fixed or random effects?. Political Sci. Res. Methods.

[28] Lajeunesse M.J. (2015). Bias and correction for the log response ratio in ecological meta-analysis. Ecology.

[29] Higgins J.P., Thompson S.G. (2002). Quantifying heterogeneity in a meta-analysis. Stat. Med..

[30] Thornton A., Lee P. (2000). Publication bias in meta-analysis: Its causes and consequences. J. Clin. Epidemiol..

[31] Peters J.L., Sutton A.J., Jones D.R., Abrams K.R., Rushton L. (2006). Comparison of two methods to detect publication bias in meta-analysis. JAMA.

[32] Duval S., Tweedie R. (2000). A nonparametric “trim and fill” method of accounting for publication bias in meta-analysis. J. Am. Stat. Assoc..

[33] Robinet C., Roques A. (2010). Direct impacts of recent climate warming on insect populations. Integr. Zool..

[34] Lehmann P., Ammunét T., Barton M., Battisti A., Eigenbrode S.D., Jepsen J.U., Kalinkat G., Neuvonen S., Niemelä P., Terblanche J.S. (2020). Complex responses of global insect pests to climate warming. Front. Ecol. Environ..

[35] Rao M.S., Manimanjari D., Rao A.C.R., Swathi P., Maheswari M. (2014). Effect of climate change on *Spodoptera litura* Fab. on peanut: A life table approach. Crop Prot..

[36] Neven L.G. (2000). Physiological responses of insects to heat. Postharvest Biol. Technol..

[37] Régnière J., Powell J., Bentz B., Nealis V. (2012). Effects of temperature on development, survival and reproduction of insects: Experimental design, data analysis and modeling. J. Insect Physiol..

[38] Jung J.M., Byeon D.H., Jung S., Lee W.H. (2019). Effect of climate change on the potential distribution of the common cutworm (*Spodoptera litura*) in South Korea. Entomol. Res..

[39] Fu X., Zhao X., Xie B., Ali A., Wu K. (2015). Seasonal pattern of *Spodoptera litura* (Lepidoptera: Noctuidae) migration across the Bohai Strait in northern China. J. Econ. Entomol..

[40] Sahu B., Pachori R., Navya R.N., Patidar S. (2020). Extent of damage by *Spodoptera litura* on cabbage. J. Entomol. Zool. Stud..

[41] Sangle P.M., Satpute S.B., Khan F.S., Rode N.S. (2015). Impact of climate change on insects. Trends Biosci..

[42] Jaworski T., Hilszczanski J. (2013). The effect of temperature and humidity changes on insects development their impact on forest ecosystems in the expected climate change. Lesn. Pr. Badaw..

