# Supplementary material for: Influence of Temperature, Humidity, and Photophase on the Developmental Stages of Spodoptera litura (Lepidoptera: Noctuidae) and Prediction of Its Population Dynamics
Source: Insects. 2025 Mar 27;16(4):355. doi: 10.3390/insects16040355 (PMC12027962; doi:10.3390/insects16040355)
Supplement: Supplementary file 1 [file insects-16-00355-s001.zip › Supplementary Material for Review.pdf]

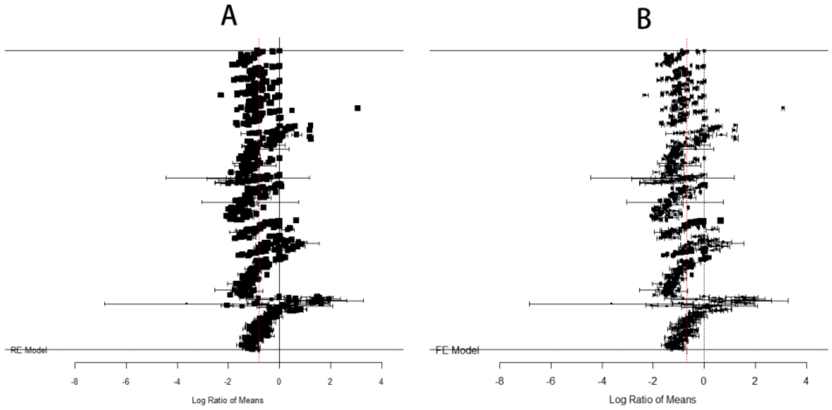

Figure S1 Comparison of effect sizes between random-effects and fixed-effects models (Panel A displays the results of the random-effects model calculation, E = -0.8077, 95% confidence interval ranging from -0.8509 to -0.7645, Q (df = 856) = 1294697.6124, $p < 0.0001$, $I^2 = 99.94\%$. Panel B shows the results of the fixed-effects model calculation, E = -0.6888, 95% confidence interval ranging from -0.6898 to -0.6878, Q (df = 856) = 1294697.6124, $p < 0.0001$, $I^2 = 99.93\%$.)

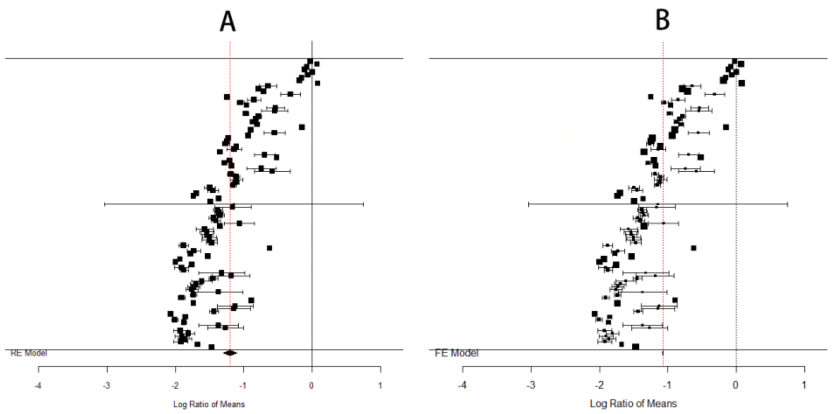

Figure S2 Comparison of effect sizes between random-effects and fixed-effects models (Panel A displays the results of the random-effects model calculation, E = -1.2022, 95% confidence interval ranging from -1.3081 to -1.0963, Q (df = 104) = 186844.6622, $p < 0.0001$, $I^2 = 99.93\%$. Panel B shows the results of the fixed-effects model calculation, E = -1.0725, 95% confidence interval ranging from -1.0752 to -1.0697, Q (df = 104) = 186844.6622, $p < 0.0001$, $I^2 = 99.94\%$.)

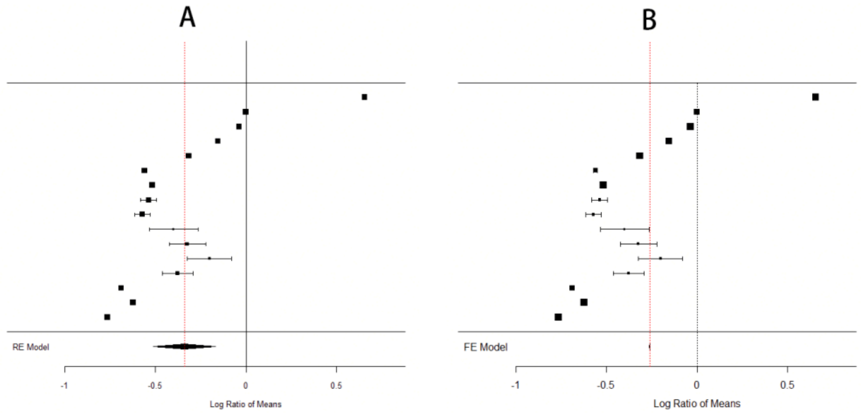

Figure S3 Comparison of effect sizes between random-effects and fixed-effects models (Panel A displays the results of the random-effects model calculation, E = -0.3387, 95% confidence interval ranging from -0.5102 to -0.1672, Q (df = 15) = 194476.1146, p < 0.0001, I^2 = 99.99%. Panel B shows the results of the fixed-effects model calculation, E = -0.2610, 95% confidence interval ranging from -0.2629 to -0.2591, Q (df = 15) = 194476.1146, p < 0.0001, I^2 = 99.99%.)

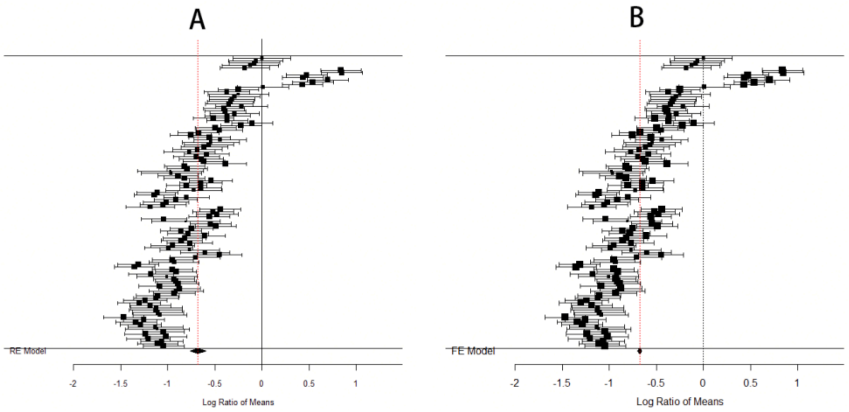

Figure S4 Comparison of effect sizes between random-effects and fixed-effects models (Panel A displays the results of the random-effects model calculation, E = -0.6795, 95% confidence interval ranging from -0.7630 to -0.5960, Q (df =120) = 1918.8339, $p < 0.0001$, $I^2 = 93.36\%$. Panel B shows the results of the fixed-effects model calculation, E =-0.6784, 95% confidence interval ranging from -0.6999 to -0.6569, Q (df =120) = 1918.8339, $p < 0.0001$, $I^2 = 93.75\%$.)

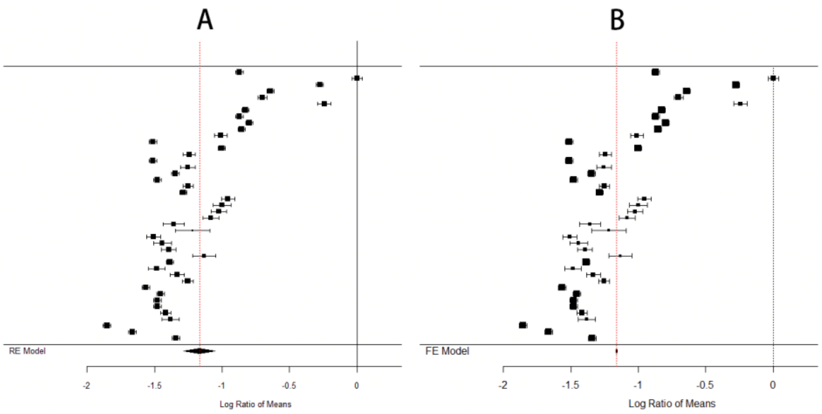

Figure S5 Comparison of effect sizes between random-effects and fixed-effects models (Panel A displays the results of the random-effects model calculation, E = -1.1675, 95% confidence interval ranging from -1.2839 to -1.0512, Q (df = 42) = 23455.7640, p < 0.0001, I^2 =99.80%. Panel B shows the results of the fixed-effects model calculation, E = -1.1675, 95% confidence interval ranging from -1.2839 to -1.0512, Q (df = 42) = 23455.7640, p < 0.0001, I^2 = 99.80%.)

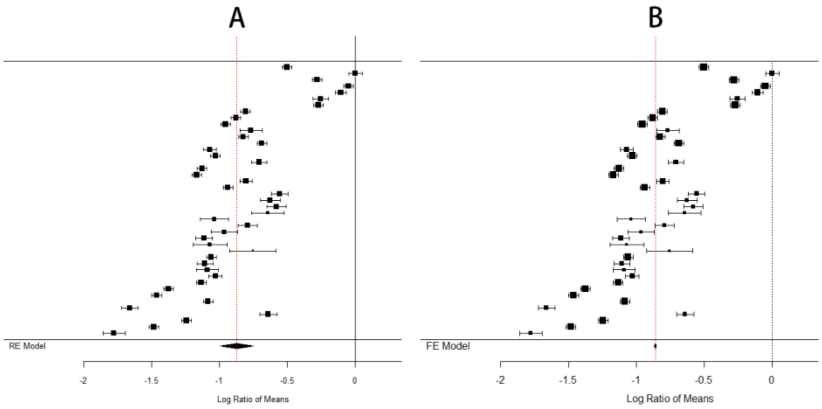

Figure S6 Comparison of effect sizes between random-effects and fixed-effects models (Panel A displays the results of the random-effects model calculation, E = -0.8725, 95% confidence interval ranging from -0.9938 to -0.7512, Q (df = 42) = 14107.8006, $p < 0.0001$, $I^2 = 99.68\%$. Panel B shows the results of the fixed-effects model calculation, E = -0.7477, 95% confidence interval ranging from -0.7595 to -0.7360, Q (df = 42) = 14107.8006, $p < 0.0001$, $I^2 = 99.70\%$.)

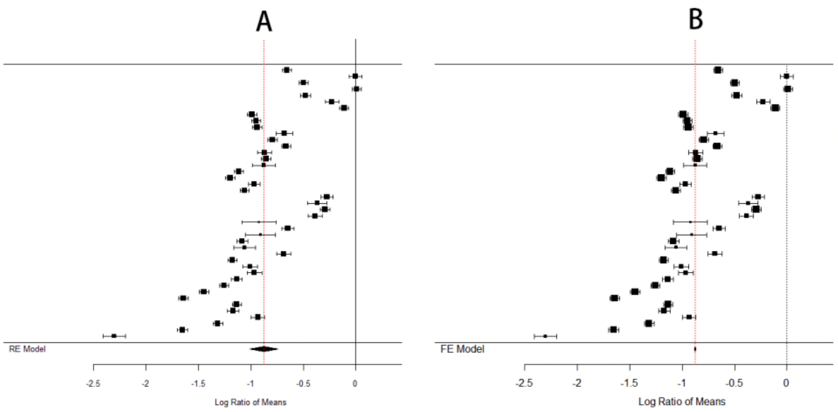

Figure S7 Comparison of effect sizes between random-effects and fixed-effects models (Panel A displays the results of the random-effects model calculation, E = -0.8763, 95% confidence interval ranging from -1.0132 to -0.7393, Q (df = 42) = 11253.4212, $p < 0.0001$, $I^2 = 99.65\%$. Panel B shows the results of the fixed-effects model calculation, E = -0.8758, 95% confidence interval ranging from -0.8839 to -0.8678, Q (df = 42) = 11253.4212, $p < 0.0001$, $I^2 = 99.63\%$.)

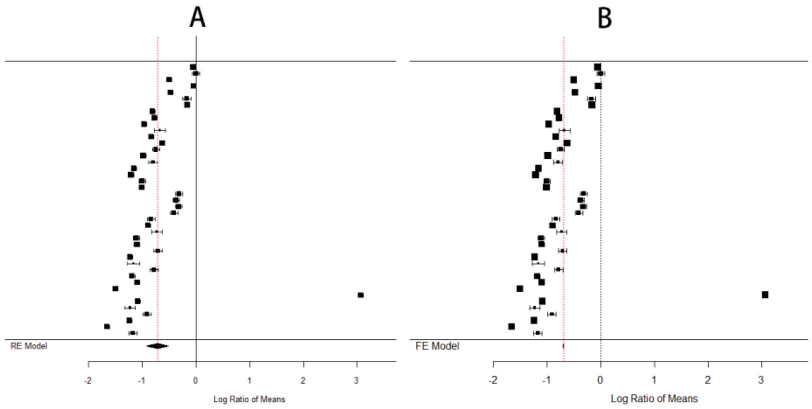

Figure S8 Comparison of effect sizes between random-effects and fixed-effects models (Panel A displays the results of the random-effects model calculation, E = -0.7200, 95% confidence interval ranging from -0.9332 to -0.5068, Q (df = 42) = 33856.4678, p < 0.0001, I^2 = 99.84%. Panel B shows the results of the fixed-effects model calculation, E = -0.6959, 95% confidence interval ranging from -0.7045 to -0.6873, Q (df = 42) = 33856.4678, p < 0.0001, I^2 = 99.88%.)

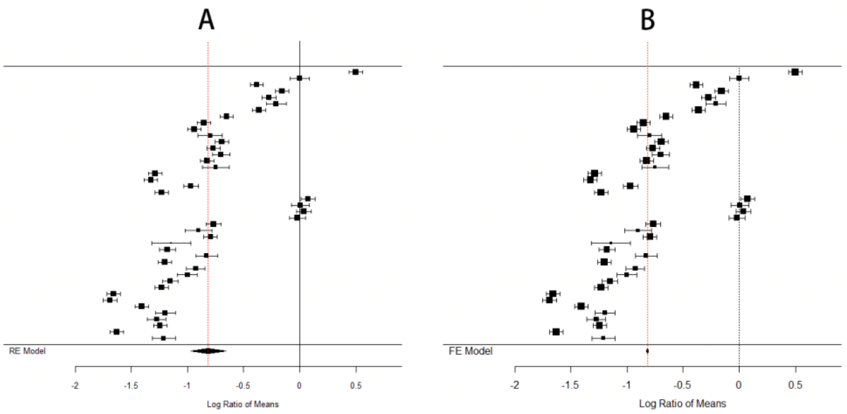

Figure 9S Comparison of effect sizes between random-effects and fixed-effects models (Panel A displays the results of the random-effects model calculation, E = -0.8143, 95% confidence interval ranging from -0.9701 to -0.6586, Q (df = 42) = 10319.3985, $p < 0.0001$, $I^2 = 99.56\%$. Panel B shows the results of the fixed-effects model calculation, E = -0.8189, 95% confidence interval ranging from -0.8293 to -0.8086, Q (df = 42) = 5289.4684, $p < 0.0001$, $I^2 = 99.59\%$.)

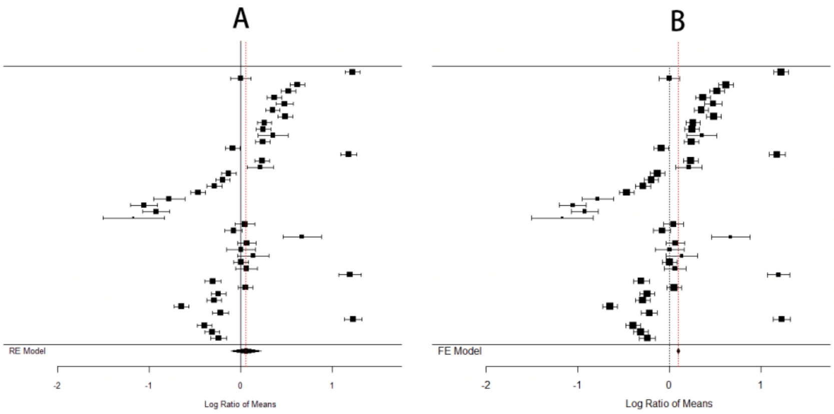

Figure S10 Comparison of effect sizes between random-effects and fixed-effects models (Panel A displays the results of the random-effects model calculation, E = 0.0597, 95% confidence interval ranging from -0.1070 to 0.2263, Q (df = 42) = 4719.5454, $p < 0.0001$, $I^2 = 99.27\%$. Panel B shows the results of the fixed-effects model calculation, E = 0.1004, 95% confidence interval ranging from 0.0863 to 0.1145, Q (df = 42) = 4719.5454, $p < 0.0001$, $I^2 = 99.11\%$.)

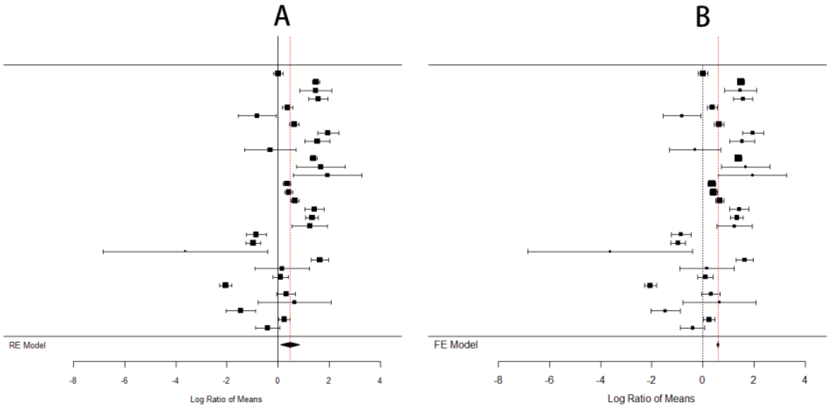

Figure S11 Comparison of effect sizes between random-effects and fixed-effects models (Panel A displays the results of the random-effects model calculation, E = 0.4685, 95% confidence interval ranging from 0.0167 to 0.0847, Q (df = 30) = 1268.4113, $p < 0.0001$, $I^2 = 98.33\%$. Panel B shows the results of the fixed-effects model calculation, E = 0.5862, 95% confidence interval ranging from 0.5393 to 0.6330, Q(df = 30) = 1268.4113, $p < 0.0001$, $I^2 = 97.63\%$.)

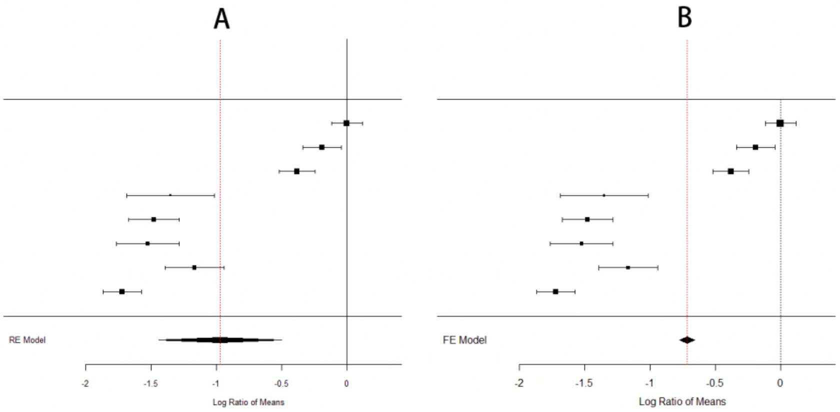

Figure S12 Comparison of effect sizes between random-effects and fixed-effects models (Panel A displays the results of the random-effects model calculation, E = -0.9705, 95% confidence interval ranging from -1.4431 to -0.4979, Q (df = 7) = 522.9586, p < 0.0001, I^2 = 98.37%. Panel B shows the results of the fixed-effects model calculation, E = -0.7167, 95% confidence interval ranging from -0.7754 to -0.6579, Q (df = 7) = 522.9586, p < 0.0001, I^2 = 98.66%.)

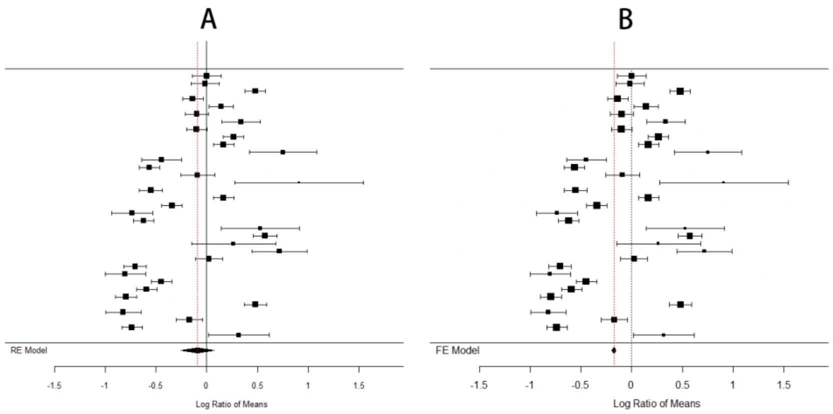

Figure S13 Comparison of effect sizes between random-effects and fixed-effects models (Panel A displays the results of the random-effects model calculation, E = -0.0920, 95% confidence interval ranging from -0.2546 to 0.0707, Q (df = 34) = 1561.5650, $p < 0.0001$, $I^2 = 98.20\%$. Panel B shows the results of the fixed-effects model calculation, E = -0.1760, 95% confidence interval ranging from -0.1975 to -0.1545, Q (df = 34) = 1561.5650, $p < 0.0001$, $I^2 = 97.82\%$.)

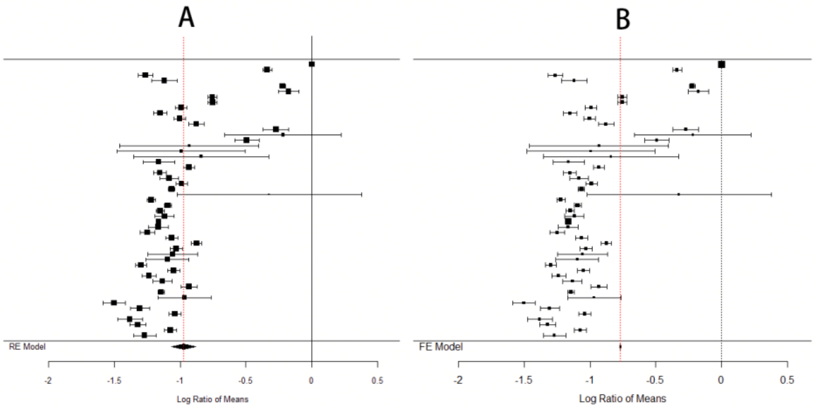

Figure S14 Comparison of effect sizes between random-effects and fixed-effects models (Panel A displays the results of the random-effects model calculation, E = -0.9734, 95% confidence interval ranging from -1.0667 to -0.8801 Q (df = 50) = 33496.1114, $p < 0.0001$, I^2 =99.64%. Panel B shows the results of the fixed-effects model calculation, E = -0.7679, 95% confidence interval ranging from -0.7732 to -0.7626, Q (df = 50) = 33496.1114, $p < 0.0001$, I^2 =99.85%.)

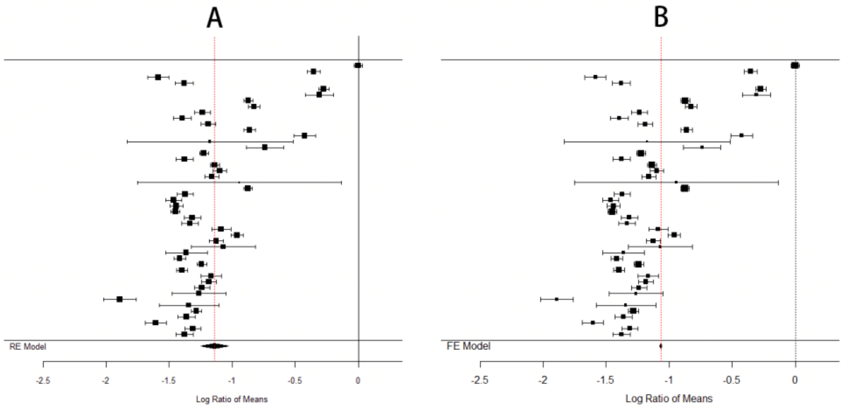

Figure S15 Comparison of effect sizes between random-effects and fixed-effects models (Panel A displays the results of the random-effects model calculation, E = -1.0654, 95% confidence interval ranging from -1.0735 to -1.0574, Q (df = 46) = 9471.6015, $p < 0.0001$, $I^2 = 99.51\%$. Panel B shows the results of the fixed-effects model calculation, E = -1.1394, 95% confidence interval ranging from -1.2488 to -1.0300, Q (df = 46) = 9471.6015, $p < 0.0001$, $I^2 = 99.44\%$.)

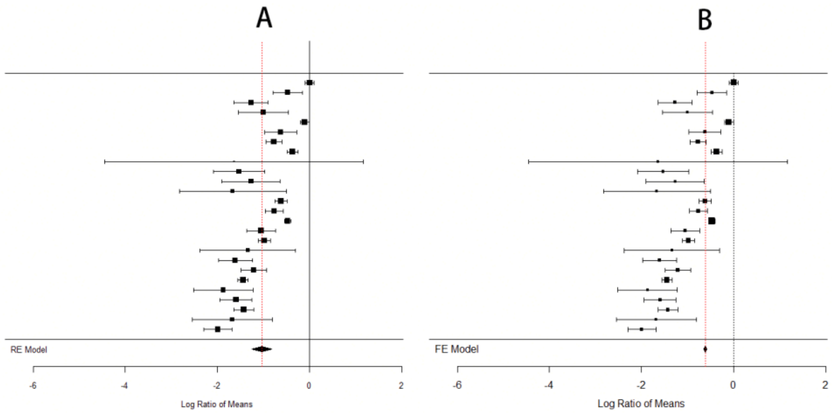

Figure S16 Comparison of effect sizes between random-effects and fixed-effects models (Panel A displays the results of the random-effects model calculation, E = -1.0392, 95% confidence interval ranging from -1.2611 to -0.8173, Q (df = 25) = 802.4040, $p < 0.0001$, $I^2 = 96.96\%$. Panel B shows the results of the fixed-effects model calculation, E = -0.6208, 95% confidence interval ranging from -0.6553 to -0.5864, Q (df = 25) = 802.4040, $p < 0.0001$, $I^2 = 96.88\%$.)

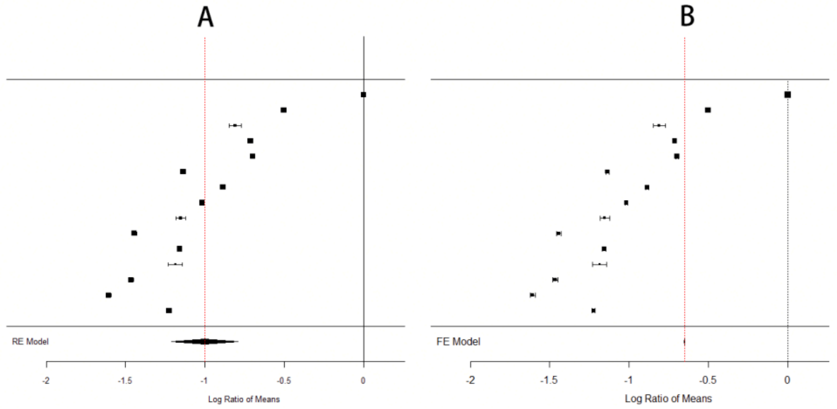

Figure S17 Comparison of effect sizes between random-effects and fixed-effects models (Panel A displays the results of the random-effects model calculation, E = -1.0001, 95% confidence interval ranging from -1.2099 to -0.7902, Q (df = 14) = 145836.8982, $p < 0.0001$, $I^2 = 99.99\%$. Panel B shows the results of the fixed-effects model calculation, E = -0.6505, 95% confidence interval ranging from -0.6529 to -0.6480, Q (df = 14) = 145836.8982, $p < 0.0001$, $I^2 = 99.99\%$.)

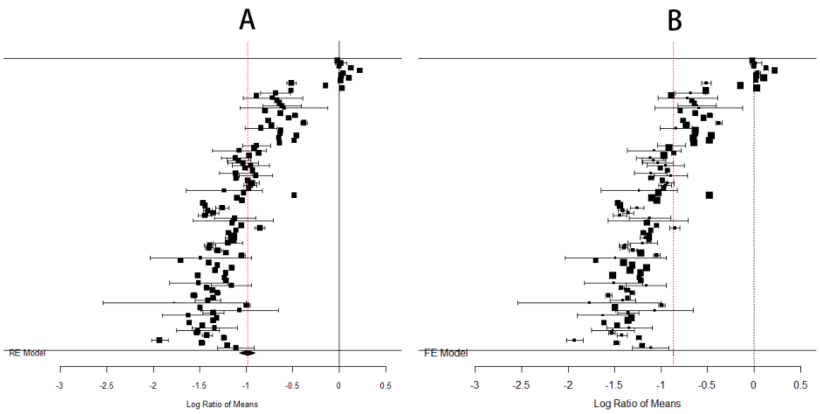

Figure S18 Comparison of effect sizes between random-effects and fixed-effects models (Panel A displays the results of the random-effects model calculation, E = -0.9870, 95% confidence interval ranging from -1.0716 to -0.9025, Q (df = 115) = 244635.1938, $p < 0.0001$, $I^2 = 99.95\%$. Panel C shows the results of the fixed-effects model calculation, E = -0.8691, 95% confidence interval ranging from -0.8710 to -0.8672, Q (df = 115) = 244635.1938, $p < 0.0001$, $I^2 = 99.95\%$.)

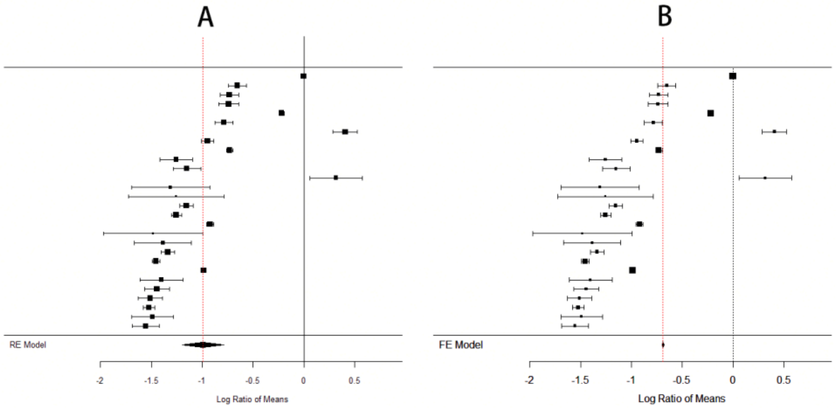

Figure S19 Comparison of effect sizes between random-effects and fixed-effects models (Panel A displays the results of the random-effects model calculation, E = -0.9917, 95% confidence interval ranging from -1.1961 to -0.7873, *Q* (df = 27) = 10909.1208, $p < 0.0001$, $I^2 = 99.75\%$. Panel C shows the results of the fixed-effects model calculation, E = -0.6874, 95% confidence interval ranging from -0.6971 to -0.6778, *Q* (df = 27) = 10909.1208, $p < 0.0001$, $I^2 = 99.75\%$.)
