# Supplementary figures and images for: Influence of Temperature, Humidity, and Photophase on the Developmental Stages of Spodoptera litura (Lepidoptera: Noctuidae) and Prediction of Its Population Dynamics
Source: Insects. 2025 Mar 27;16(4):355. doi: 10.3390/insects16040355 (PMC12027962; doi:10.3390/insects16040355)

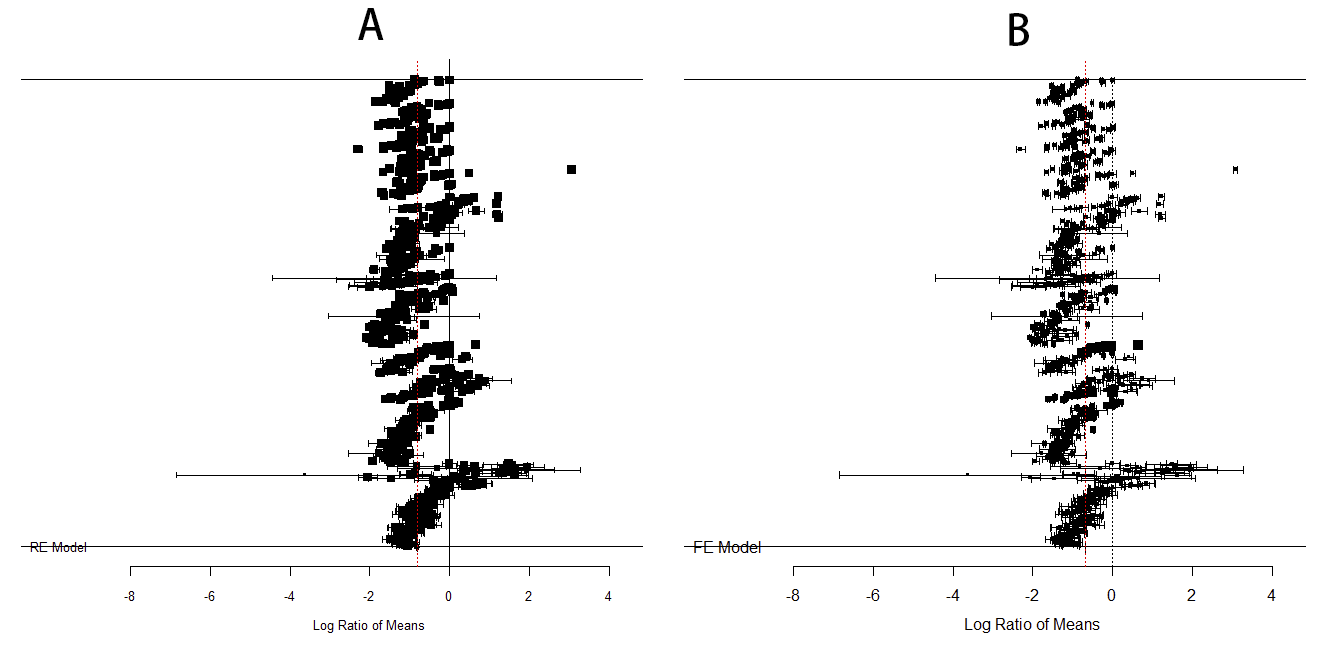

Supplement: Supplementary file 1 [file insects-16-00355-s001.zip › Fig S1.tif]

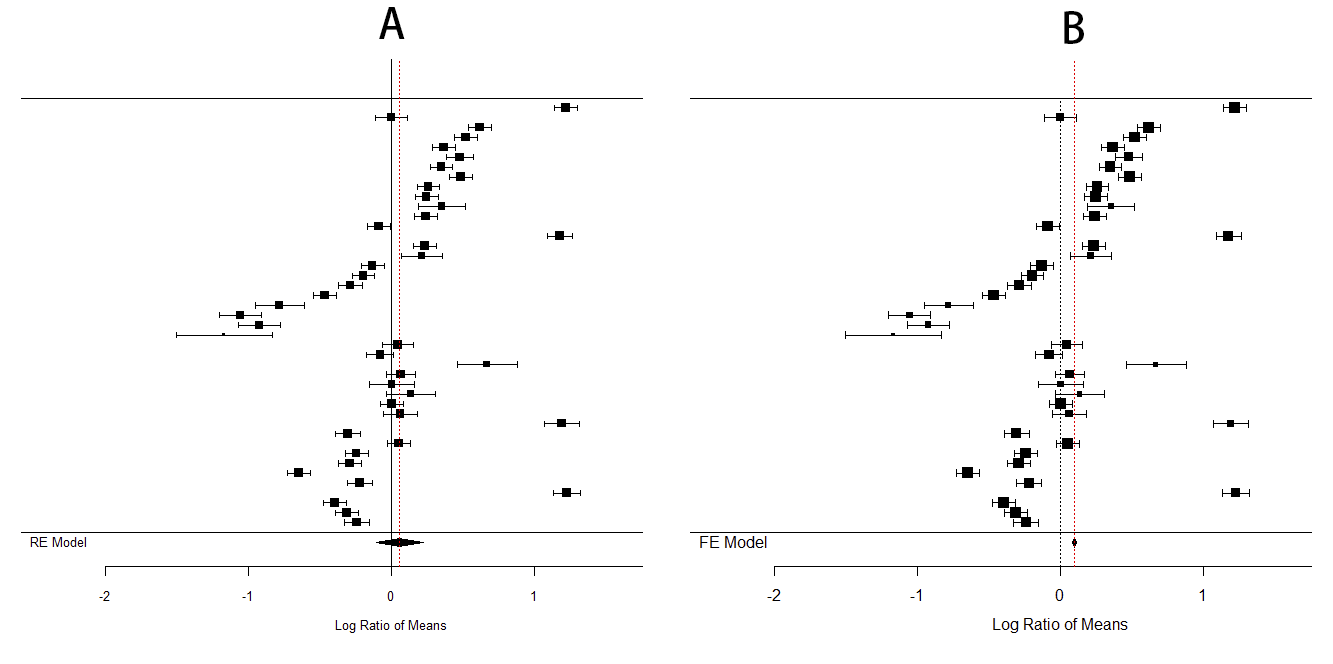

Supplement: Supplementary file 1 [file insects-16-00355-s001.zip › Fig S10.tif]

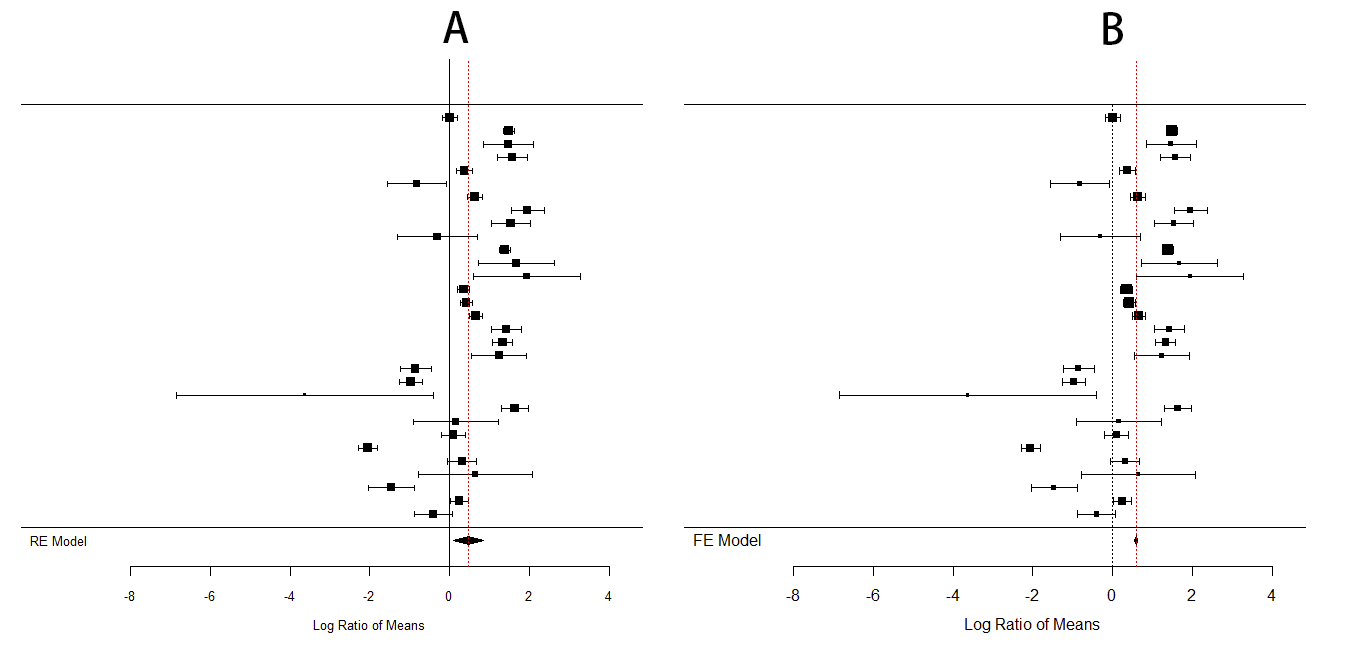

Supplement: Supplementary file 1 [file insects-16-00355-s001.zip › Fig S11.tif]

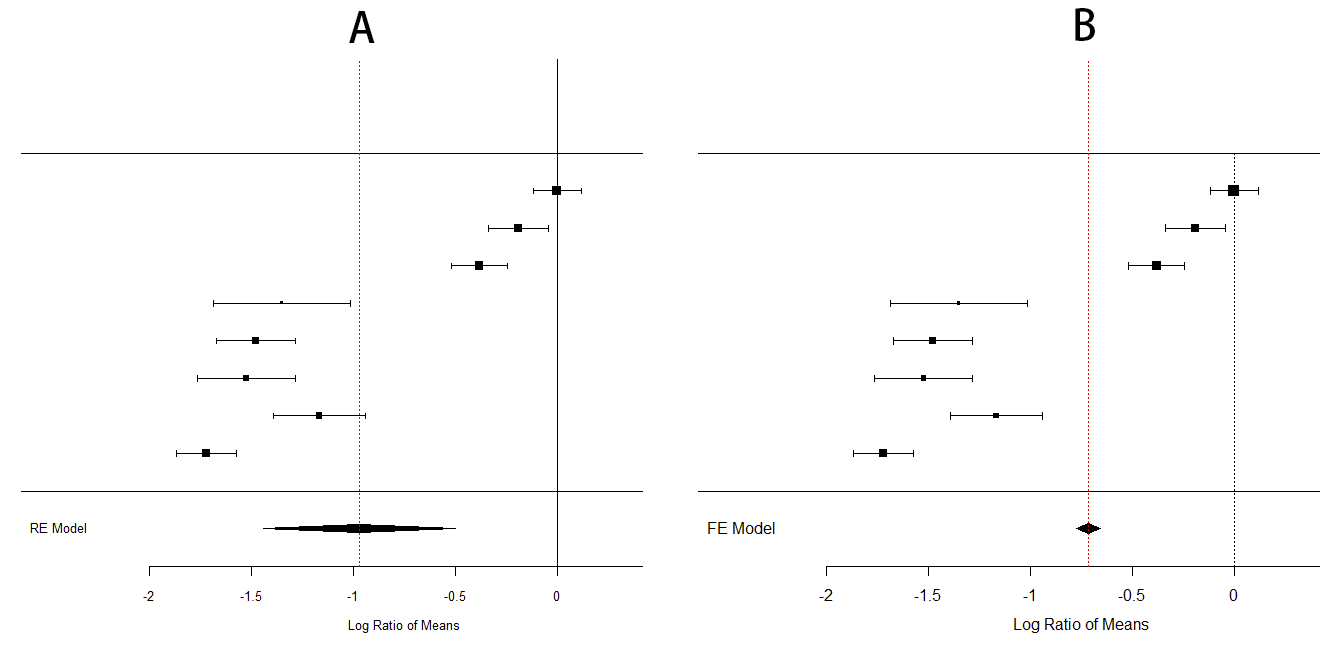

Supplement: Supplementary file 1 [file insects-16-00355-s001.zip › Fig S12.tif]

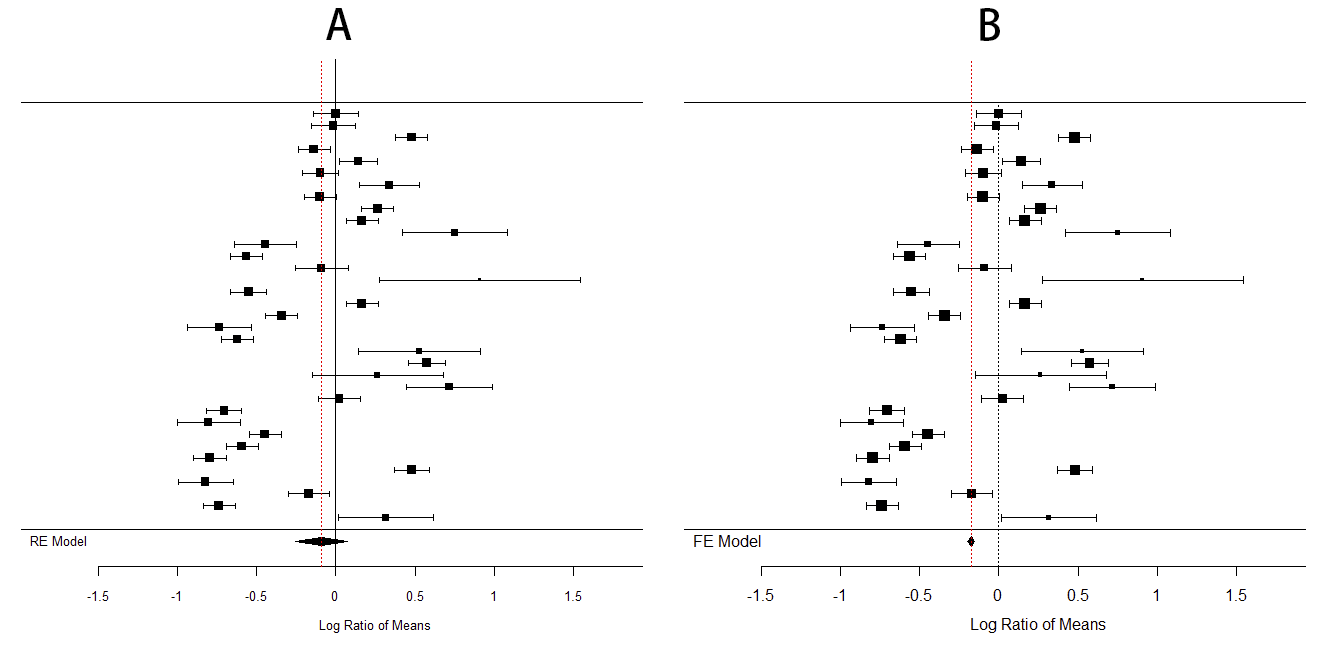

Supplement: Supplementary file 1 [file insects-16-00355-s001.zip › Fig S13.tif]

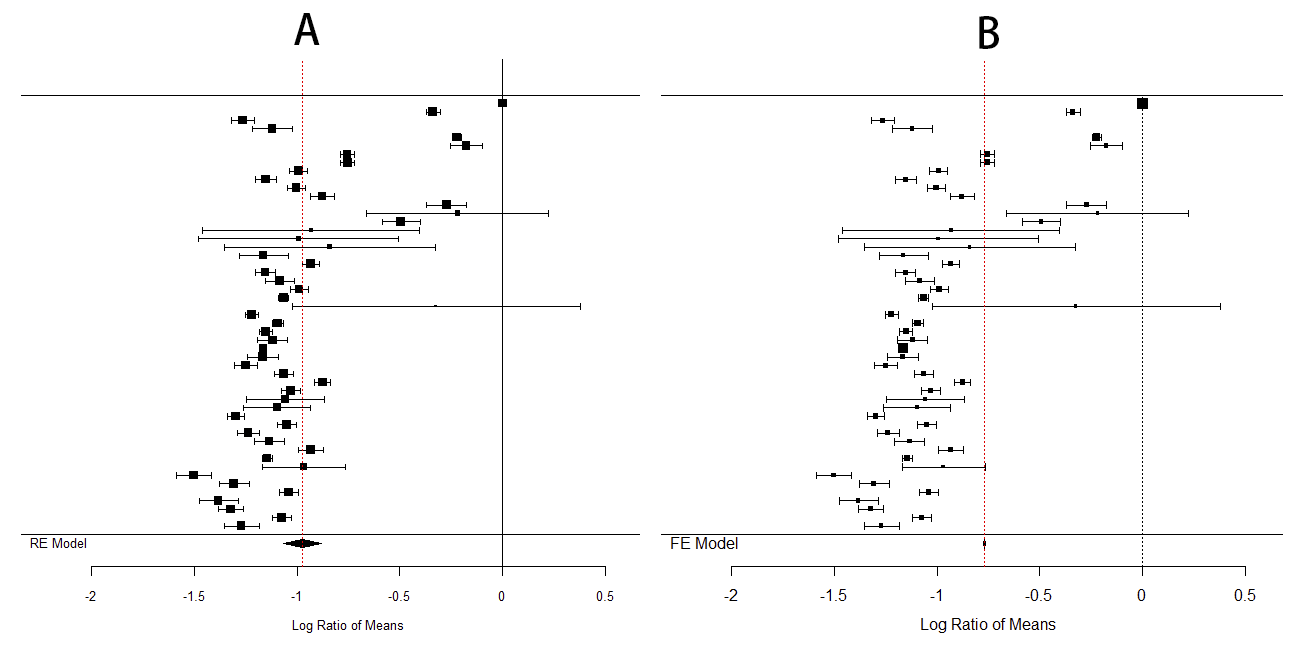

Supplement: Supplementary file 1 [file insects-16-00355-s001.zip › Fig S14.tif]

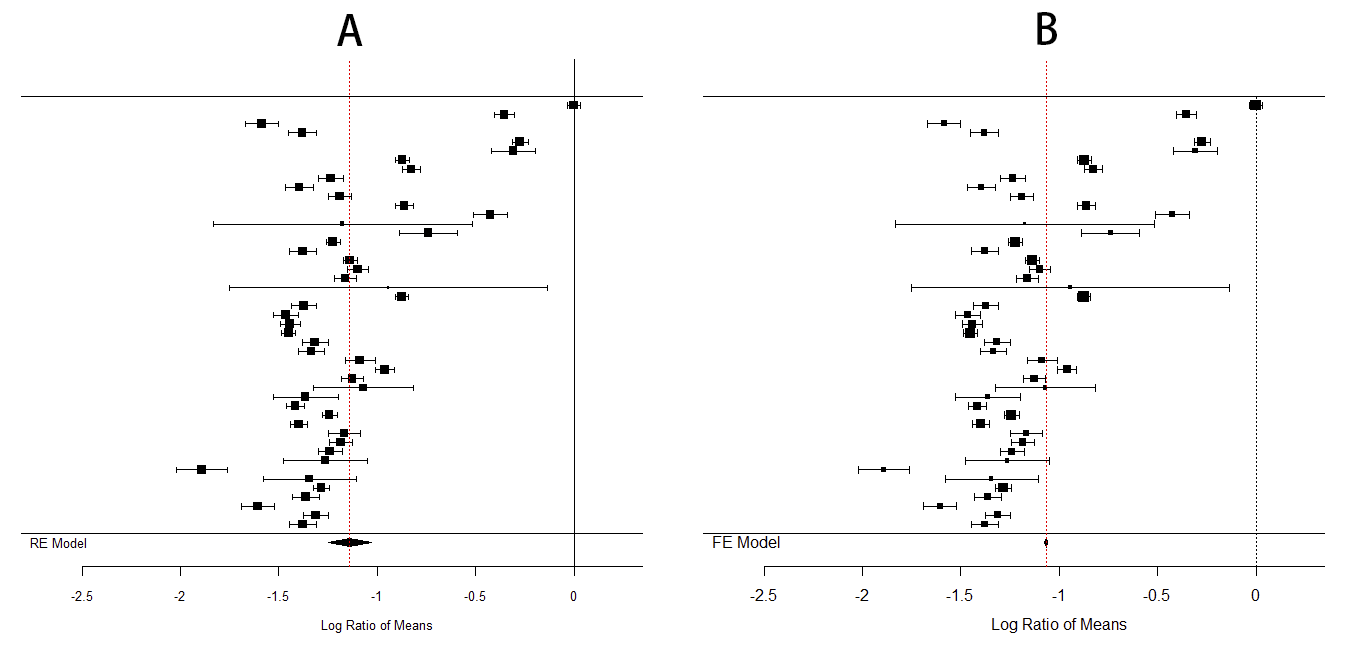

Supplement: Supplementary file 1 [file insects-16-00355-s001.zip › Fig S15.tif]

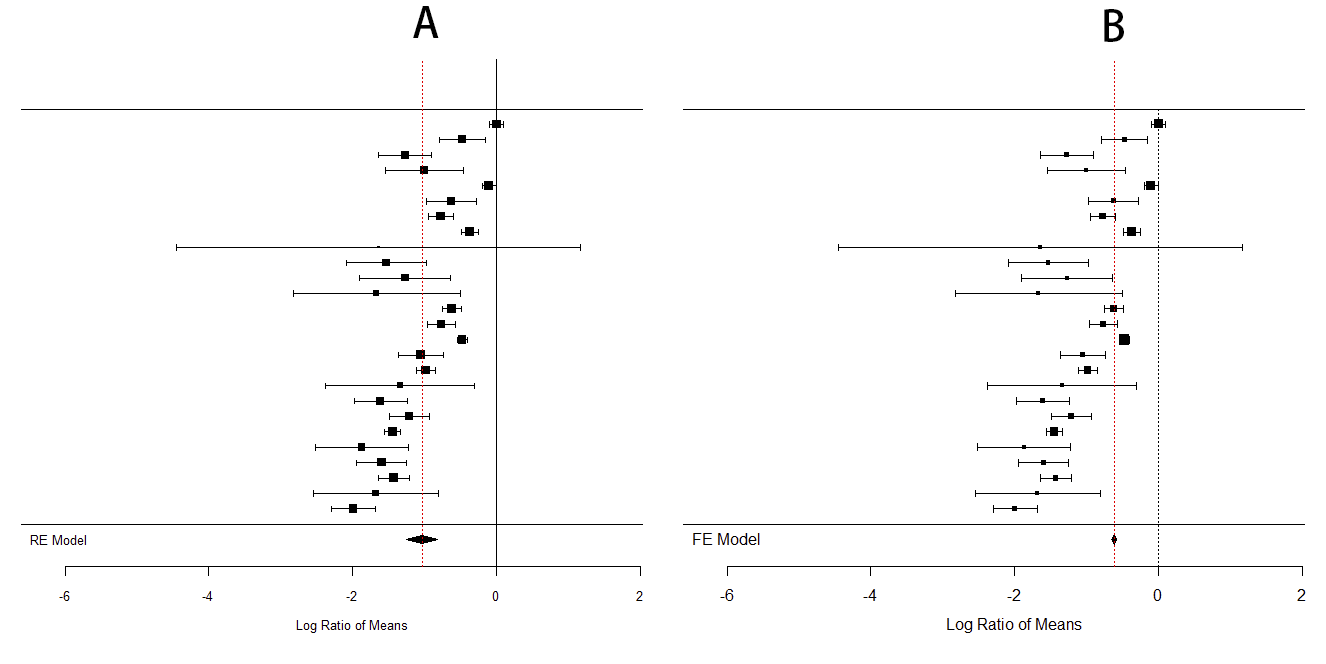

Supplement: Supplementary file 1 [file insects-16-00355-s001.zip › Fig S16.tif]

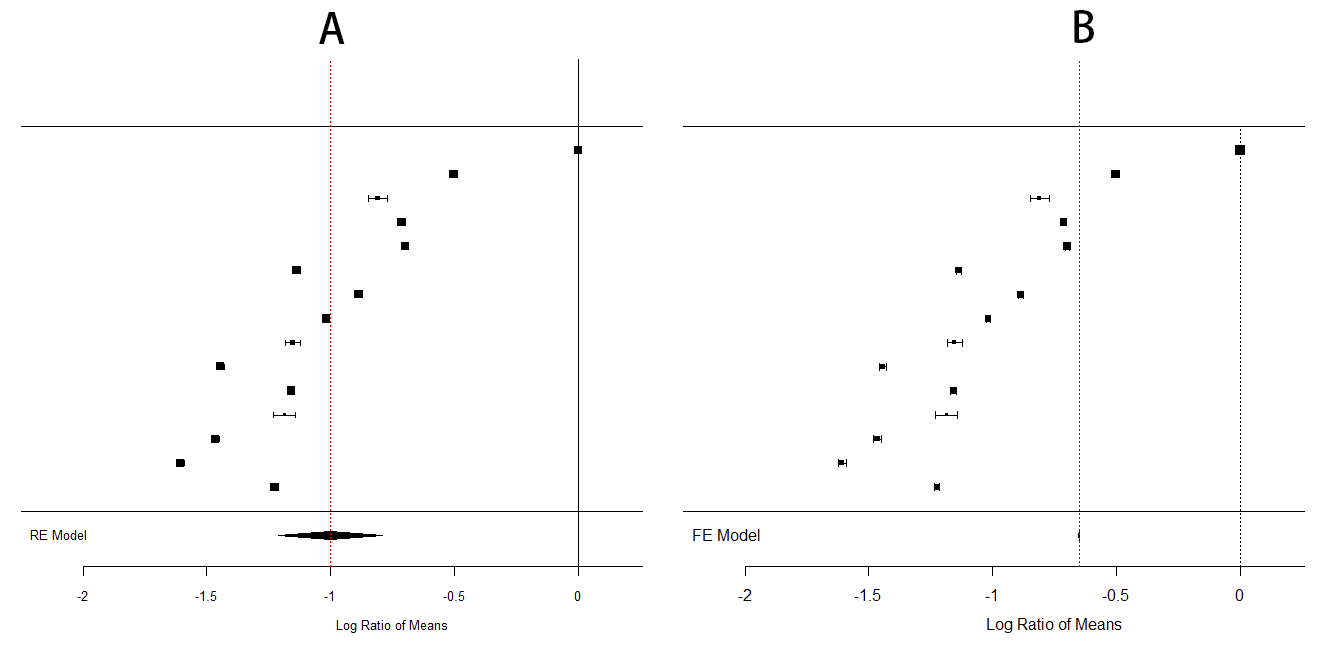

Supplement: Supplementary file 1 [file insects-16-00355-s001.zip › Fig S17.tif]

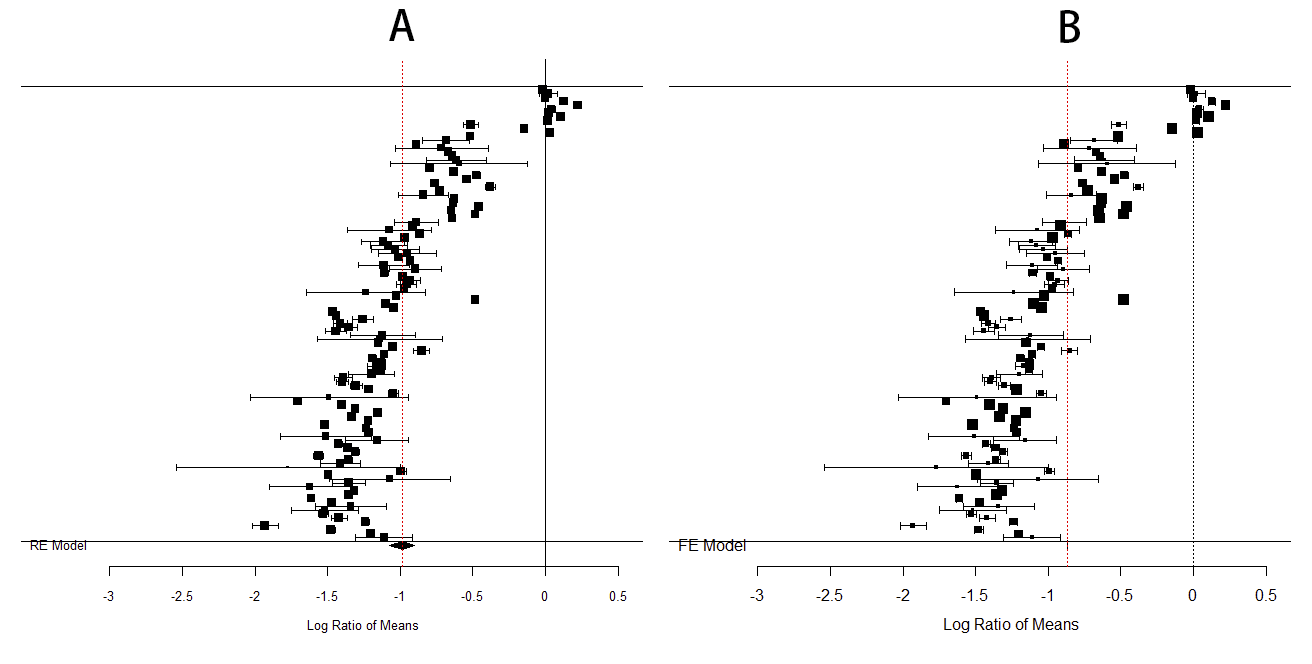

Supplement: Supplementary file 1 [file insects-16-00355-s001.zip › Fig S18.tif]

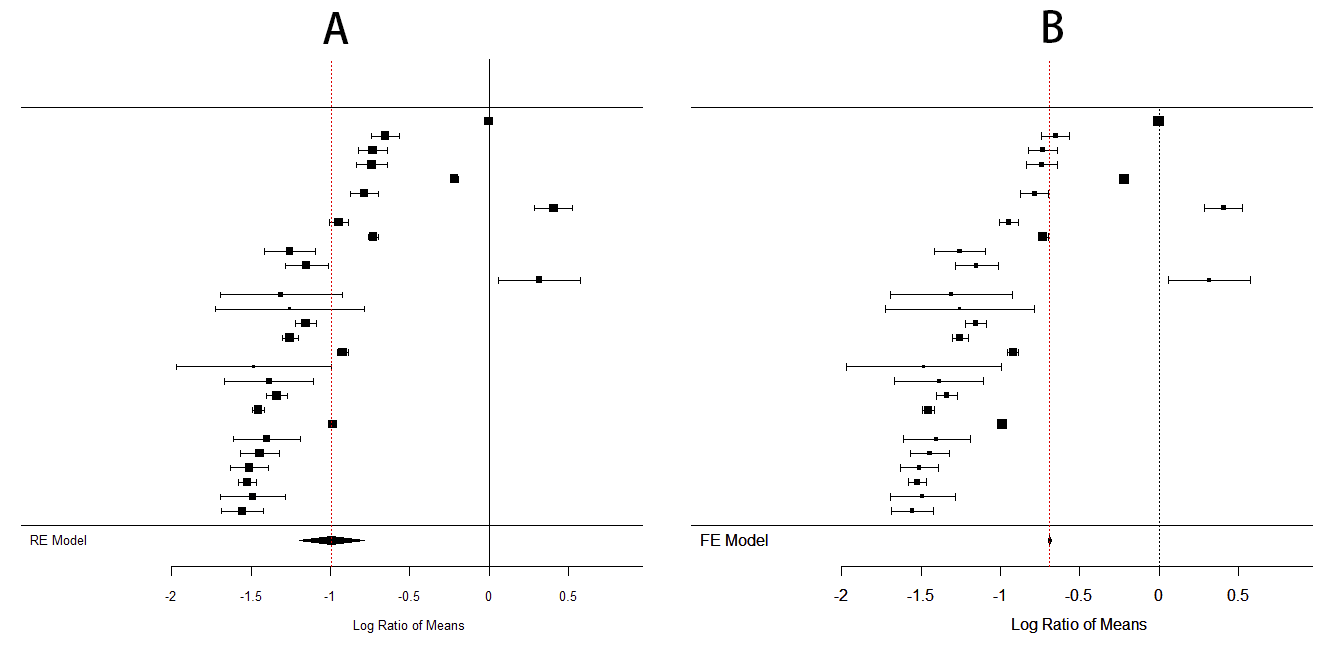

Supplement: Supplementary file 1 [file insects-16-00355-s001.zip › Fig S19.tif]

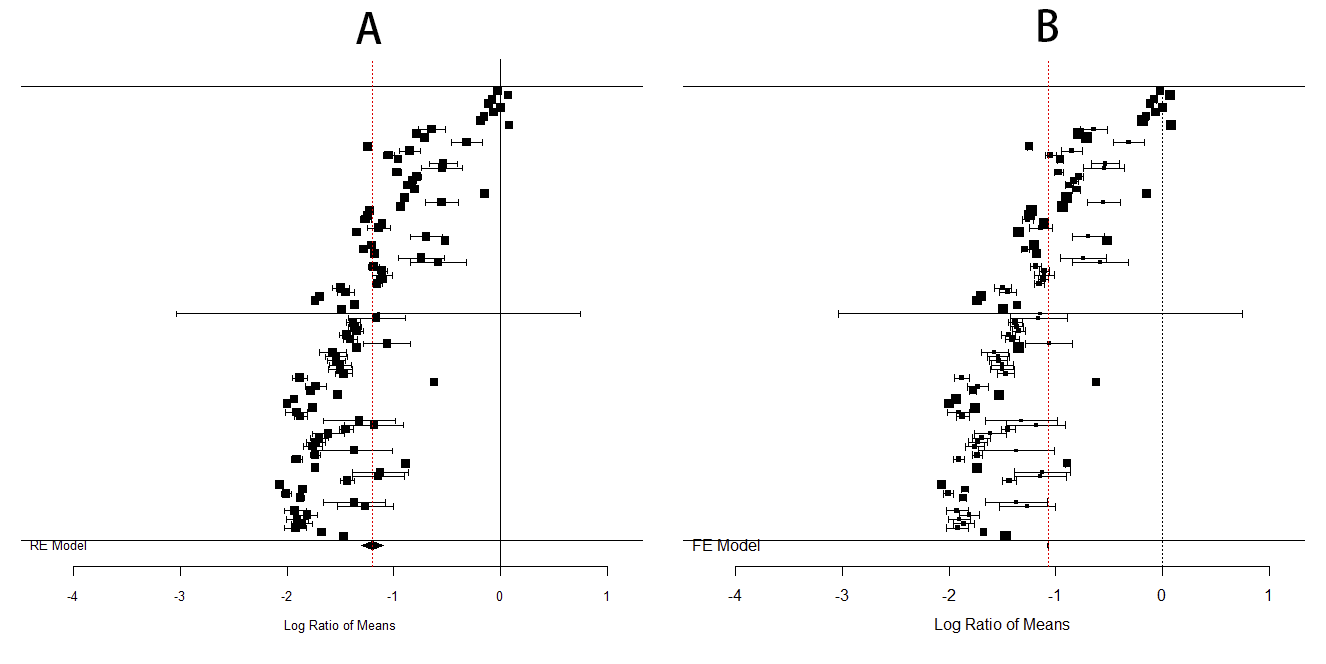

Supplement: Supplementary file 1 [file insects-16-00355-s001.zip › Fig S2.tif]

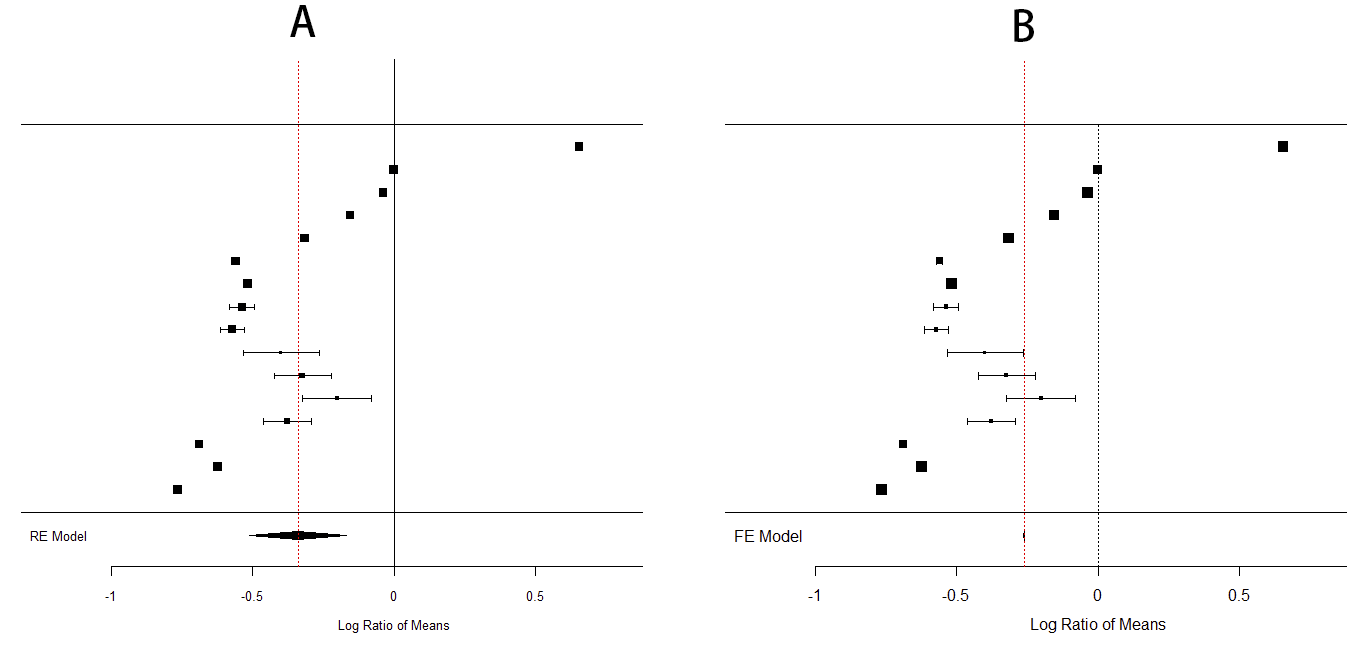

Supplement: Supplementary file 1 [file insects-16-00355-s001.zip › Fig S3.tif]

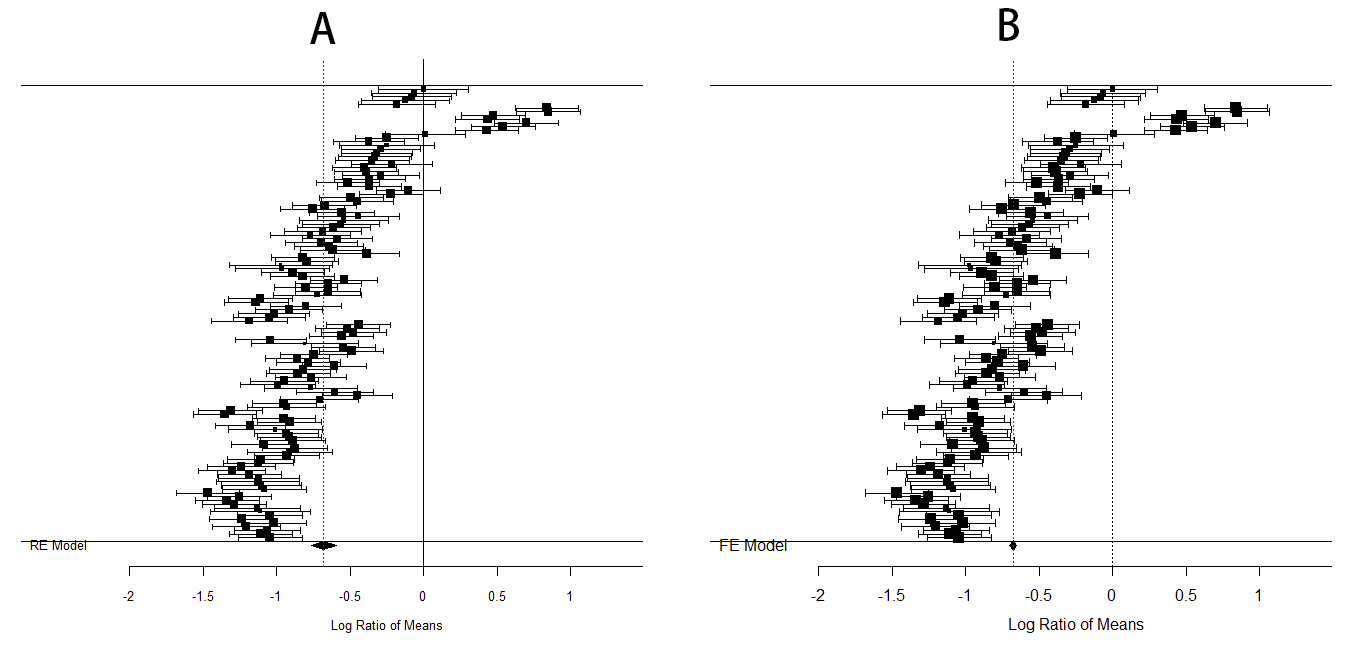

Supplement: Supplementary file 1 [file insects-16-00355-s001.zip › Fig S4.tif]

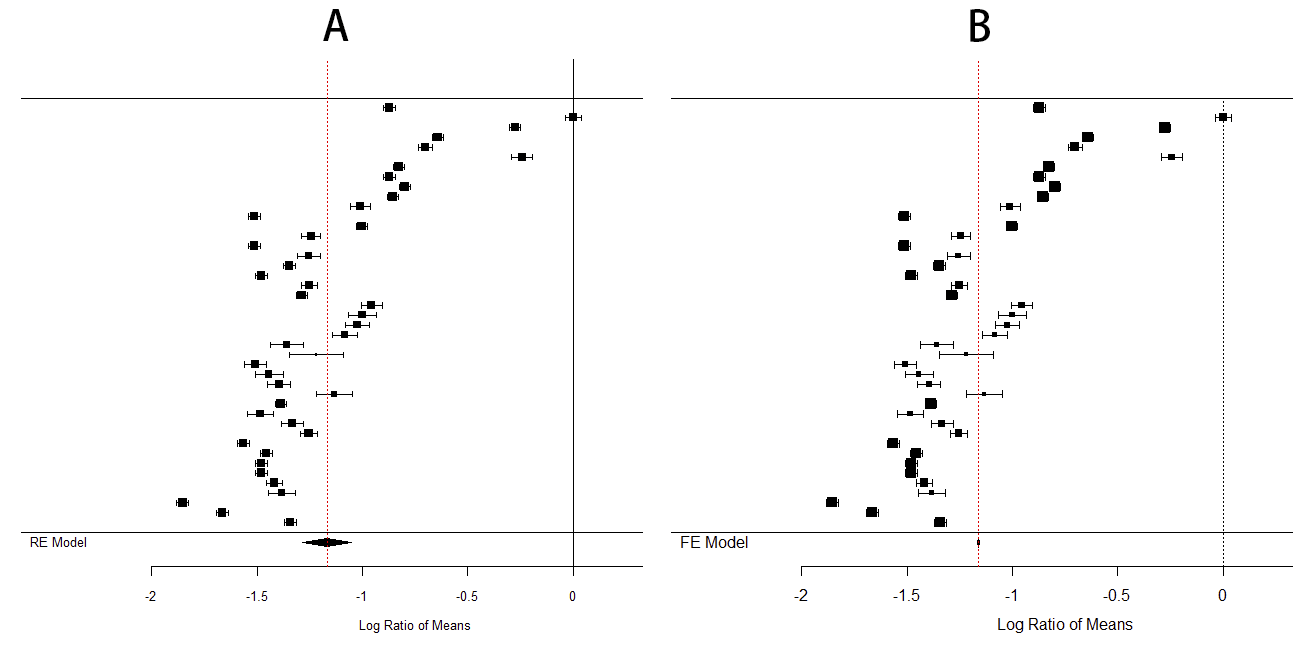

Supplement: Supplementary file 1 [file insects-16-00355-s001.zip › Fig S5.tif]

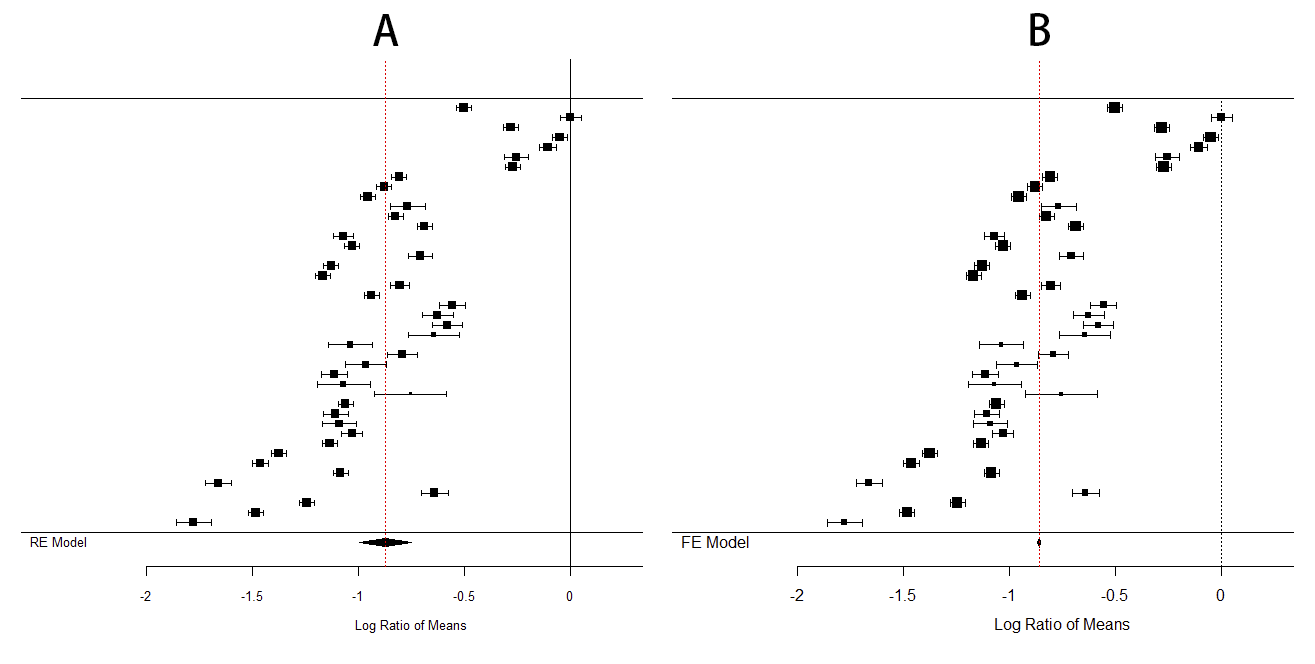

Supplement: Supplementary file 1 [file insects-16-00355-s001.zip › Fig S6.tif]

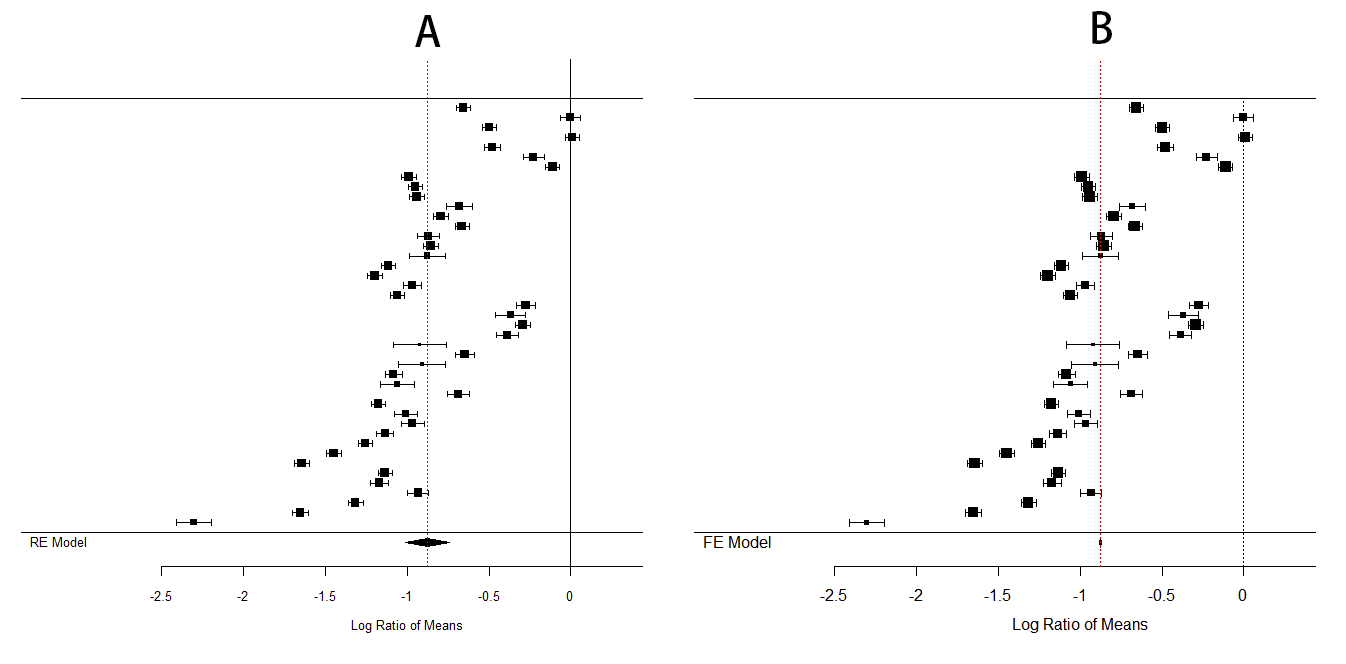

Supplement: Supplementary file 1 [file insects-16-00355-s001.zip › Fig S7.tif]

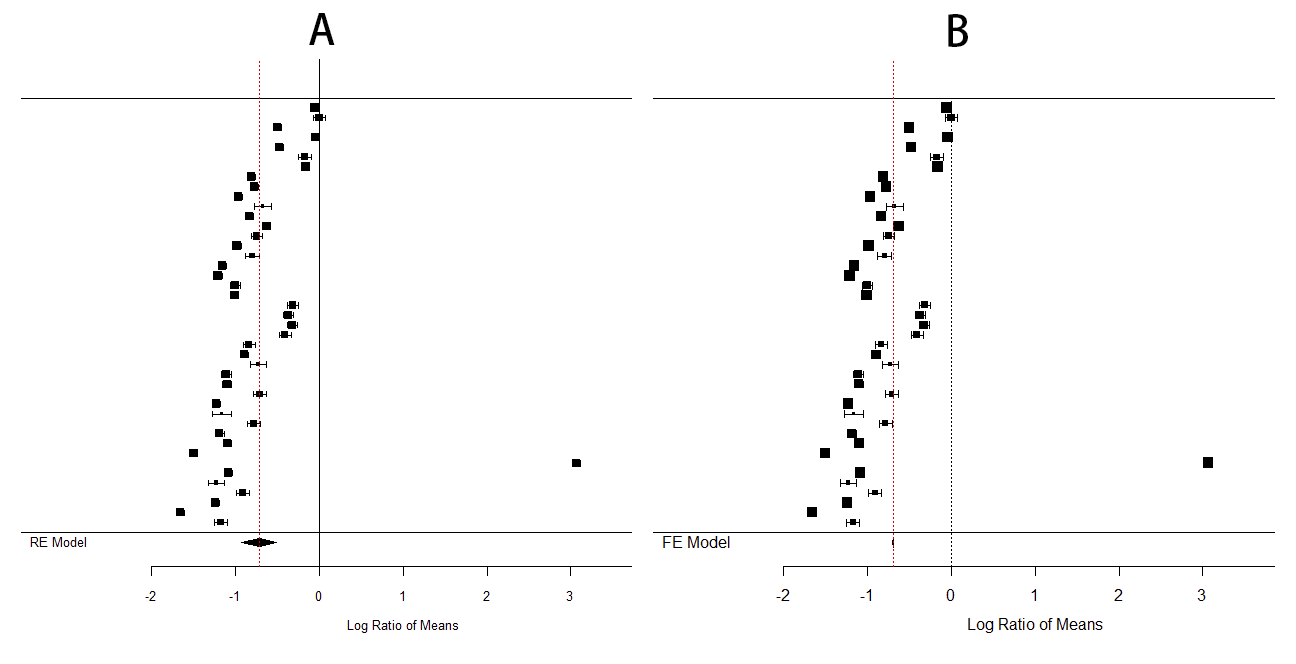

Supplement: Supplementary file 1 [file insects-16-00355-s001.zip › Fig S8.tif]

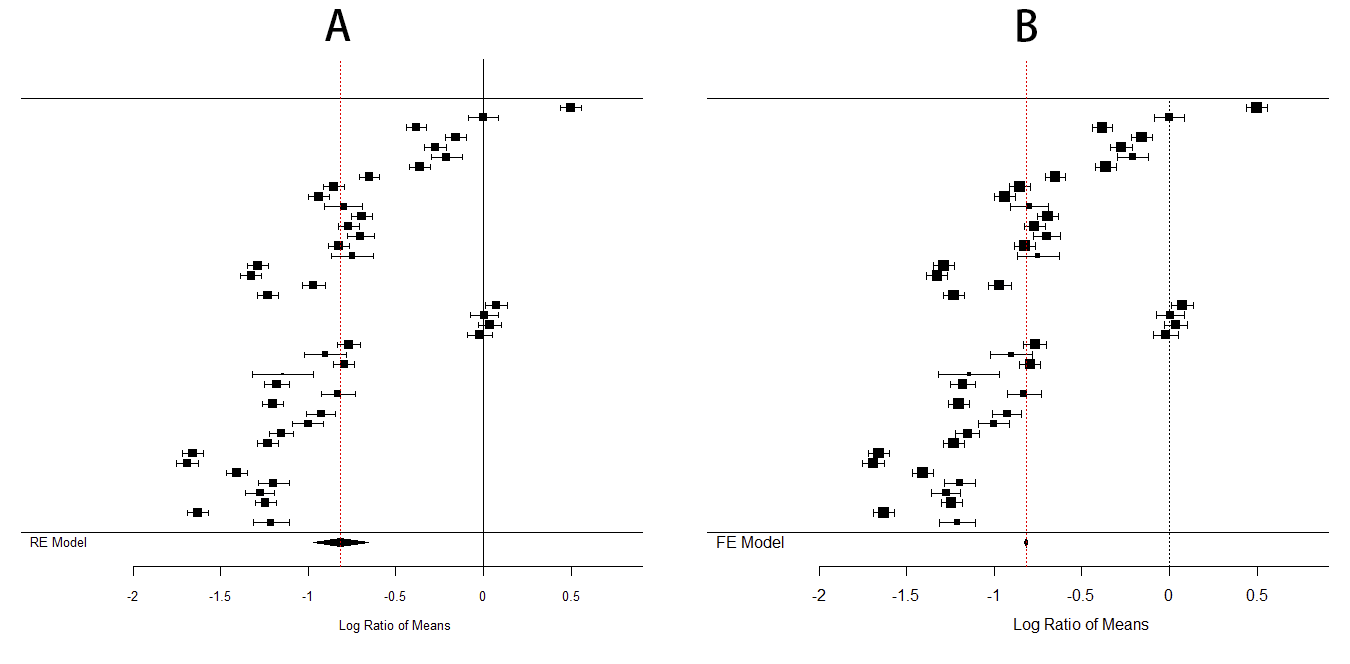

Supplement: Supplementary file 1 [file insects-16-00355-s001.zip › Fig S9.tif]
